# N6-methyloxyadenine-mediated detoxification and ferroptosis confer a trade-off between multi-fungicide resistance and fitness

**DOI:** 10.1128/mbio.03177-23

**Published:** 2024-01-31

**Authors:** Borui Zhang, Zhiwen Wang, Sicong Zhang, Shan Zhong, Ye Sun, Xili Liu

**Affiliations:** 1 China Agricultural University, Beijing, China; 2 Sanya Institute of China Agricultural University, Sanya, China; 3 State Key Laboratory of Crop Stress Biology for Arid Areas, Northwest A&F University, Yangling, China; Universidade de Sao Paulo, Ribeirao Preto, Sao Paulo, Brazil

**Keywords:** DNA N6-methyloxyadenine, fitness penalty, detoxification, ferroptosis

## Abstract

Multi-fungicide resistance (MFR) is a serious environmental problem, which results in the excessive use of fungicides. Fitness penalty, as a common phenomenon in MFR, can partially counteract the issue of resistance due to the weakened vigor of MFR pathogens. Their underlying mechanism and relationship remain unexplained. By Oxford Nanopore Technologies sequencing and dot blot, we found that N6-methyloxyadenine (6mA) modification, the dominate epigenetic marker in *Phytophthora capsici*, was significantly altered after MFR emerged. Among the differently methylated genes, *PcGSTZ1* could efficiently detoxify SYP-14288, a novel uncoupler, through complexing the fungicide with glutathione and induce MFR. Interestingly, *PcGSTZ1* overexpression was induced by elevated 6mA levels and chromatin accessibility to its genomic loci. Moreover, the overexpression led to reactive oxygen species burst and ferroptosis in SYP-14288-resistant mutants, which enhanced the resistance and induced fitness penalty in *P. capsici* through triggering low energy shock adaptive response. Furthermore, this study revealed that the 6mA-PcGSTZ1-ferroptosis axis could mediate intergenerational resistance memory transmission and enabled adaptive advantage to *P. capsici*. In conclusion, the findings provide new insights into the biological role of 6mA as well as the mechanisms underlying the trade-off between MFR and fitness. These could also benefit disease control through the blockade of the epigenetic axis to resensitize resistant isolates.

**IMPORTANCE:**

N6-methyloxyadenine (6mA) modification on DNA is correlated with tolerance under different stress in prokaryotes. However, the role of 6mA in eukaryotes remains poorly understood. Our current study reveals that DNA adenine methyltransferase 1 (DAMT1)-mediated 6mA modification at the upstream region of GST zeta 1 (GSTZ1) is elevated in the resistant strain. This elevation promotes the detoxification uncoupler and induces multifungicide resistance (MFR). Moreover, the overexpression led to reactive oxygen species burst and ferroptosis in SYP-14288-resistant mutants, which enhanced the resistance and induced fitness penalty in *Phytophthora capsici* through triggering low energy shock adaptive response. Furthermore, this study revealed that the 6mA-PcGSTZ1-ferroptosis axis could mediate intergenerational resistance memory transmission and enabled adaptive advantage to *P. capsici*. Overall, our findings uncover an innovative mechanism underlying 6mA modification in regulating PcGSTZ1 transcription and the ferroptosis pathway in *P. capsici*.

## INTRODUCTION

Fungicides have been used to control diseases for over 160 years. The evolution of resistance poses an ongoing threat to environment and human health after excessive use of fungicides (1). *Phytophthora capsici* is a destructive plant oomycete pathogen that causes root, fruit, and foliar diseases in more than 70 vegetable crops, including pepper, tomato, eggplant, and cucurbit plants (2). However, fungicide resistance has developed in many oomycete species, including *P. capsici* (3, 4). Currently, the mechanism of fungicide resistance is primarily limited to two categories: (i) mutation and overexpression of the target protein and (ii) active efflux or metabolic breakdown of the fungicide (3). The first mechanism is commonly emerged toward fungicides with the same target by reducing the binding or by increasing the available target of the fungicide (5). Fungicide metabolism and excretion are typically simultaneous, which markedly reduce the active substances in the target organisms and usually lead to multi-fungicide resistance (MFR) (6–8). Compared to target mutation-related resistance, MFR is more harmful for plant disease control since many fungicides are rendered ineffective (9). However, the upstream regulator of metabolism/excretion-related genes remains unclear.

As a general phenomenon observed in fungicide-resistant pathogens, fitness penalty, also known as resistance cost, is an evolutionary trade-off of gaining resistance (8), especially in MFR pathogens (9), reflected by slower growth rate, lower sporulation ability, and lower virulence. Fitness penalty is closely related to resistance development. It may be due to mutations causing the loss/decrease of the function in the target gene (10, 11). In addition, high-level expression of resistance-related genes can affect the normal development. The extra energetic costs may trigger fitness penalties, especially in organisms with MFR (8).

DNA methylation is one of the fundamental epigenetic markers in both eukaryotes and prokaryotes and is imperative in many processes, including transposon silencing, gene expression regulation, and epigenetic memory maintenance (12, 13). The most studied form of DNA methylation is 5-methylcytosine (5mC), which is the prevalent DNA modification in mammals and plants (14). However, 5mC is barely detectable or absent in some eukaryotic organisms, including *Caenorhabditis elegans*, *Drosophila melanogaster*, *Saccharomyces cerevisiae,* and two important *Phytophthora* species, *Phytophthora infestans* and *Phytophthora sojae* (15). Comparatively, another prominent DNA methylation pattern newly discovered in 2016, N6-methyloxyadenine (6mA), is distributed extensively in the prokaryotic organisms, *P. infestans* and *P. sojae*, and is present in mammals, nematodes, and fungi (15). DNA methyltransferases, such as Dams, MT-A70s, or DNA adenine methyltransferases (DAMTs), are responsible for modulating 6mA by transferring the methyl group of S-adenosy-L-methionine (SAM) to the substrate deoxynucleotide (16). They contribute to prokaryotic immunity against phages and invade genetic elements through a restriction-modification system that discriminates between the host genome and invader DNA (17). The distribution of 6mA in genomic loci could strongly affect its function in eukaryotes. For example, in *Chlamydomonas* and fungi, 6mA is enriched around the transcription start sites (TSSs) of actively expressed genes, suggesting that 6mA may be an active mark for gene expression (16), while 6mA appears to suppress transcription on the X chromosome in mice (18). As 6mA modification is highly enriched in gene-sparse regions and depleted around TSSs, it may be involved in the adaptive evolution in *P. infestans* and *P. sojae* (15). Moreover, 6mA possibly performs a role during the early stages of mating and infection in *P. infestans* and *P. sojae*, respectively (15, 19). Nevertheless, more evidences are required to prove the function of 6mA in *Phytophthora*, and some researchers believe that DNA 6mA has no function due to its low abundance in most eukaryotes (20). Therefore, the existence of 6mA in *P. capsici* and its exact biological role in *Phytophthora* warrants further investigation. As an important epigenetic regulator, whether it participates in regulating fungicide resistance-related genes and fitness penalty is an interesting research topic.

5mC-associated drug resistance has been reported in *Escherichia coli* (21), *Mycobacterium tuberculosis* (22), and *Enterobacteriaceae* strains (23) through transcriptome regulation. In mammals, DNA methylation changes are associated with resistance to docetaxel in breast cancer cells and platinum in epithelial ovarian cancer cells (24, 25). Unlike 5mC, 6mA-mediated resistance has rarely been studied. 'Ma et al. found that 6mA mediates resistance to mitochondrial inhibitors (MIs) in *Caenorhabditis elegans* and promotes the alleviation of mitochondrial stress in progeny (26, 27). In another study, METTL4-mediated mitochondrial DNA (mtDNA) 6mA was found to play an important role in mammalian responses under hypoxia (28). Moreover, 6mA gives a survival advantage of antibiotic stress in *E. coli* (29). However, previous studies mainly focused on global 6mA landscape changes; exactly how the 6mA-associated regulatory axis participates in resistance development and, furthermore, how 6mA balances fungicide resistance and fitness penalty remain unknown.

As the main factors that metabolize xenobiotics, glutathione S-transferases (GSTs) are a class of phase II detoxification enzymes that catalyze the conjugation of glutathione (GSH) to endogenous or exogenous electrophilic compounds (30) and participate in drug resistance in many species (31). GST zeta 1 (GSTZ1), an important member of the GST superfamily, is involved in the catabolism of phenylalanine/tyrosine and catalyzes the isomerization of maleylacetoacetate to fumarylacetoacetate (32). The role of *GST* genes in insecticide resistance has been widely reported (33, 34). However, how to modulate *GST* expression during resistance development and the role of GST in fungicide resistance are undetermined.

As a side effect, GSTZ overexpression enhances the sorafenib-induced ferroptosis in hepatocellular carcinoma cells (35). Ferroptosis is a newly described programmed cell death characterized by an iron-dependent accumulation of lipid peroxides to lethal amounts by downregulating glutathione peroxidase 4 (GPx4), accumulating iron and lipid reactive oxygen species (ROS) (36, 37). Thus, lipid ROS production produced by the changes in mitochondrial metabolic function could participate in the execution of ferroptosis (38). Recent studies have shown that mitochondrial complex I inhibition triggers a mitophagy-dependent ROS increase leading to ferroptosis in melanoma cells (39). Another electron transport chain complex inhibitor, sorafenib, was found to generate ROS during its working period (40). As a counter-defense, strategies to suppress ROS levels and reductive redox states in sorafenib-resistant hepatocellular carcinoma cells developed (40). As over 25% of commercial fungicides are MIs (Fungicide Resistance Action Committee [FRAC], https://www.frac.info/home), whether and how ferroptosis mediates resistance against these fungicides is worth investigating. Meanwhile, as fitness penalties were commonly observed in MI-resistant microbes (8), whether they are directly triggered by ferroptosis is a worthwhile research topic.

MIs, including quinone outside inhibitors (QoIs) and succinate dehydrogenase inhibitors (SDHIs), increasing resistance occurs mainly by amino acid substitution in target proteins of plant pathogens (41). Apart from QoIs and SDHIs, uncouplers are another type of MIs and are commonly used as medicines and fungicides. Uncouplers are classified as ion transporters, which discharge the proton gradient by transporting hydrogen ions in the inner mitochondrial membrane (42). As a result, ATP synthetase cannot be activated due to the lack of proton gradient, and the oxidative phosphorylation is “uncoupled” from the electron transport chain (43, 44). Despite having been widely used for several decades, their resistance mechanism remains enigmatic (3). In our previous work, we demonstrated that SYP-14288, a novel uncoupler and analog of fluazinam that is highly effective with low toxicity (Fig. S1) (3), could efficiently induce oxidation and phosphorylation uncoupling in *P. capsici* and shows a good control efficiency on 31 tested fungi and oomycetes, presenting as a novel fungicide with great market potential (45). Meanwhile, *P. capsici* could develop high resistance against SYP-14288 (resistance fold >100), and no mutations, but changes of gene expression levels were found in four genes in the mitochondrial electron transport chain (3). Interestingly, like the plants in which DNA methylation could alter their tolerance to high temperature (46), SYP-14288-resistant isolates also exhibited a better adaptability in high temperature, which has been rarely observed in other fungicide-resistant pathogens. This implies that the mechanism of uncoupler resistance is unique. However, the biological roles of DNA modifications and their associations with uncoupler resistance are unclear.

In this study, it was revealed that 6mA dominated in *P. capsici* genome, and it was identified as an essential epigenetic adaptation to fungicides including uncouplers. PcDAMT1 plays a key role in resistance development, and 176 genes were differentially expressed and methylated between resistant and sensitive isolates. Among them, PcGSTZ1 could efficiently detoxify SYP-14288 through chelation, inducing MFR in *P. capsici*. Furthermore, overexpression of *PcGSTZ1* led to ROS burst and ferroptosis in SYP-14288-resistant mutants, which subsequently enhanced the resistance and induced fitness penalty in *P. capsici*. The 6mA-PcGSTZ1-ferroptosis regulatory axis is involved in MFR acquisition and intergenerational resistance memory transmission.

## MATERIALS AND METHODS

### *P. capsici*, *E. coli,* and plant cultivation

*P. capsici* isolate JA8 was isolated from an infected chili pepper collected in Gansu, China in 2012. SYP-14288-resistant mutants RJA1 and RJA2 were generated by SYP-14288 adaptation using JA8 as the parental isolate. *P. capsici* reference isolate LT1534 was maintained in the lab. All *P. capsici* were routinely cultured on solid potato dextrose agar (PDA) agar medium at 25°C in darkness. *E. coli* strains DH5α and the *dam* mutant HST04 were cultured on Luria broth (LB) medium at 37°C in darkness. Chili pepper cultivar Xichengdaniujiao was grown in garden soil at 25°C in a greenhouse.

### Dot blot assay

Genomic DNA (gDNA) of *P. capsici* was extracted using a TIANGEN DNAsecure Plant kit (Beijing, China) and RNA was removed by RNase treatment and column chromatogram. Equal amounts of gDNA were denatured at 95°C for 5 min and chilled on ice for 10 min. DNA were spotted onto Amersham Hybond-N^+^ membranes (GE Healthcare, Beijing, China), and further dried at 37°C for 30 min. DNA was then crosslinked under UV for 5 min. The membrane was blocked in 5% milk phosphate-buffered saline with Tween 20 (PBST) for 1 h and incubated with 6mA antibody (sysy, 202003) or 5mC antibody (Abcam, ab73938) in 5% milk PBST overnight at 4°C. After a PBST wash, the membrane was incubated with horseradish peroxidase (HRP)-conjugated goat anti-mouse IgG secondary antibody (Proteintech, Beijing, China) for 1 h and treated with ECL substrate (CWBIO, Jiangsu, China). After washing, the signal was detected by Tanon 5200 (Shanghai, China). For input quantification, the same membrane was incubated with 0.1% methylene blue solution for 15 min and washed using tris-buffered saline with Tween 20 (TBST) buffer three times. Relative 6mA abundance was quantified (integrated signal density anti-6mA/integrated signal density input DNA) using ImageJ (NIH image to ImageJ: 25 years of image analysis.).

### Quantification of 6mA and D3-6mA by ultra-performance liquid chromatography ripple-quadrupole mass spectrometry (UPLC-QqQ-MS/MS)

The detection of 6mA and D3-6mA by UPLC-QqQ-MS/MS was performed as previously described (26). The gDNA was dissolved in 40 µL nuclease-free H_2_O, digested with DNase I (NEB, Beijing, China) at 37°C for 2 h, denatured at 95°C for 5 min, chilled on ice, and then incubated with 4 µL 100 mM NH_4_OAc (pH 5.3) and 1 µL of nuclease P1 (Merck, Shanghai, China) at 42°C for 6 h. After the addition of 0.1 × volume of 1 M NH_4_HCO_3_ and 1 µL alkaline phosphatase (Merck, Shanghai, China), the samples were incubated at 37°C for 6 h. The final solution was centrifuged at 13,000 rpm for 30 min, and 5 µL was injected into LC–MS/MS.

The detection of dA, 6mA, and D3-6mA was performed on ACQUITY UPLC-QqQ system (Waters) and the ACQUITY UPLC BEH C18 column (1.7 µm, 2.1 mm × 100 mm, Waters). A gradient elution system consisting of mobile phase A (acetonitrile) and B (5 mM NH_4_Ac) was used. The flow rate was 0.3 mL/min. Separations were performed with the following gradient: 100% B (0–2 min), 85–100% B (2–4 min), 85% B (4–8 min), and 85%–100% B (8–12 min). The ion mass transition was 266.1–150.1 for 6mA, 252.1–136.0 for dA, and 269.1–153.1 for D3-6mA.

### Quantification of SYP-14288 and GSH-SYP by high-performance liquid chromatography (HPLC)-MS/MS

SYP-14288 and GSH-SYP detection by HPLC-MS/MS was performed as previously described (47). Sensitive strain JA8 and SYP-14288-resistant mutants RJA1 were routinely cultured on potato dextrose broth (PDB) with 1.5 µg/mL SYP-14288. After 15 h, the hyphae samples from the PDB were obtained using a vacuum pump filter device. Freeze-dried mycelium (0.05 g) was added to 10 mg adsorbent C18 with 1.5 mL acetonitrile for extraction, vortexed for 1 min, sonicated for 10 min, and centrifuged at 4,000 rpm for 10 min. Supernatant was filtered through a 0.22 µm microporous membrane for HPLC-MS/MS analysis.

The detection of SYP-14288 and GSH-SYP was achieved using HPLC-Q-TOF (Agilent 1260/6520) and the ACQUITY UPLC BEH C18 column (1.7 µm, 2.1 mm × 100 mm, Agilent). A gradient elution system consisting of mobile phase A (methanol) and B (H_2_O) was used. The flow rate was 0.3 mL/min. Separations were performed with the following gradient: 65% B (0–2 min), 65%–95% B (2–17 min), 95%–95% B (17–20 min), 95%–65% B (20–20.1 min), and 65%–65% B (20.1–28 min). Data were analyzed with Agilent MassHunter Quantitative Analysis software v.B.07.00 (Agilent technologies) to confirm peak identities.

### *Dpn*I-dependent methylation assay and *E. coli*-based *in vivo* methylation assay

PcDAMT1, PcDAMT2, PcDAMT3, EscDAM, and green fluorescent protein (GFP) were expressed using TNT SP6 High-Yield Wheat Germ Protein Expression System (Promega, Beijing, China) according to the manufacturer protocol. The *in vitro* methylation assay was performed as previously described (48). Briefly, 1 µg N6-methyladenine-free lambda DNA was methylated by the purified recombinant proteins (4 µg or 20 µg) in methylation buffer (20 mM Tris-HCl, pH 8.0, 50 mM NaCl, 7 mM 2-mercaptoethanol, 1 mM EDTA, 0.1 mg/mL bovine serum albumin [BSA], and 50 µM SAM). After incubation at 37°C for 1 h and 65°C for 15 min to stop the reaction, the productive DNA was further processed to a *Dpn*I-dependent methylation assay . The DNA was digested by 5 U *Dpn*I at 37°C for 1 h. Digestion was stopped by heat inactivation by incubating at 80°C for 20 min. One percent agarose gel electrophoresis was used to check the digestion. For the *in vivo* methylation assay, *PcDAMT1*, *PcDAMT2*, *PcDAMT3*, *EscDAM,* and *GFP* were cloned into *pEASY*-Blunt E1 Expression vector (Transgen, Beijing, China), and the recombinant plasmids were expressed in 6mA-deficient *E. coli* HST04 strains (*dam*-, *dcm−*), respectively. After verification by western blot, DNA of the transformants were extracted by the cetyltrimethylammonium bromide (CTAB) method and subjected to dot blot assays performed as previously described. All the primers used in this study were listed in Table S2.

### RNA extraction, quantitative reverse transcription PCR (qRT-PCR), and RNA-seq

Total RNA of 2-day-old *P. capsici* hyphae were isolated using Eastep Super Total RNA Extraction Kit (Promega, Shanghai, China) according to the manufacturer protocol. RNA concentration and purity was measured using NanoDrop 2000 (Thermo Fisher Scientific, Wilmington, DE). RNA integrity was assessed using the RNA Nano 6000 Assay Kit of the Agilent Bioanalyzer 2100 system (Agilent Technologies, CA, USA). RNA reverse transcription was conducted using SuperRT cDNA Synthesis Kit (CWBIO, Jiangsu, China). Quantitative RT-PCR was performed using UltraSYBR One Step RT-qPCR kit (CWBIO, Jiangsu, China) at the qTOWER2.2 Real-Time qPCR System (Jena, Beijing, China). RNA-seq was performed by Biomarker (Beijing, China). A total amount of 1 µg RNA per sample was used as input material for the RNA sample preparations. Sequencing libraries were generated using NEBNext Ultra RNA Library Prep Kit for Illumina (NEB, USA) following manufacturer recommendations. The clustering of the index-coded samples was performed on a cBot Cluster Generation System using TruSeq PE Cluster Kit v4-cBot-HS (Illumina) according to the manufacturer instructions. After cluster generation, the library preparations were sequenced on an Illumina platform and paired-end reads were generated.

### Oxford Nanopore Technologies sequencing (ONT-seq) for analyzing the 6mA profiles in *P. capsici*

The ONT-seq was performed by Biomarker (Beijing, China) according to the protocol provided by ONT (49). Briefly, gDNA was extracted and treated with RNase A overnight. The samples were quality controlled and quantified by Nanodrop, Qubit, and 0.35% agarose gel electrophoresis. The gDNA was broken into 8 kb fragments by g-TUBE, and the sequencing library was constructed using SQK-LSK109 ligation kit (ONT, Shanghai, China) following the manufacturer protocol. The sequencing of the libraries was performed in Illumina. The analysis results of ONT-seq were performed using the BMKCloud platform (https://www.biocloud.net/). Basecall was performed by Guppy (50), and the clean reads were obtained after quality control and filtration. Then, clean reads were subjected to bioinformatics analysis.

### High-throughput sequence data analysis

Clean reads obtained from ONT-seq data were mapped to *P. capsici* v 11.0 using minimap2. The 6mA loci were detected by the re-squiggle algorithm and alternative model using tombo. The loci with more than 10× depth were selected for further analysis. The methylation level in repeat regions was predicted by RepeatMasker. The 6mA motif was analyzed using meme algorithm and the ZOOPS model. Differentially methylated loci (DMLs) were screened using SMART2 (*P* < 0.05, level of difference > 0.1), and their annotations were performed by ChIPseeker. The enrichment analysis (gene ontology [GO] and Kyoto Encyclopedia of Genes and Genomes [KEGG]) of the DML-associated genes were performed using clusterProfiler.

RNA-seq data were mapped to *P. capsici* v 11.0 using HISAT2 (HISAT: a fast spliced aligner with low memory requirements) and assembled using String Tie. The mapped data were visualized using Integrative Genomics Viewer. Quantification of gene expression levels were estimated by fragments per kilobase of transcript per million fragments mapped (fpkm). The formula is shown as follows: fpkm = cDNA fragments / (mapped fragments [millions] × transcript length [kb]). Differential expression analysis of two conditions/groups was performed using the edgeR. The resulting *P*-values were adjusted using the Benjamini-Hochberg approach for controlling the false discovery rate. Genes with an adjusted *P-*value (false discovery rate [FDR]) <0.05 found by edgeR were assigned as differentially expressed. GO and KEGG enrichments of the differentially expressed genes (DEGs) were performed by GOseq R and KABAS, respectively.

To combine the analyses of ONT-seq and RNA-seq, genes were divided into five groups according to their expression level as low (0.1 ≤ fpkm < 1), medium_low (1 ≤ fpkm < 10), medium (10 ≤ fpkm < 100), medium_high (100 ≤ fpkm < 1,000), and high (fpkm ≥1,000). Their methylation level was counted by a region-wise method. The relationship of methylation and expression was further confirmed by correlation analysis. The genes to be classified into the four different profiles (hypermethylated/upregulated, hypermethylated/downregulated, hypomethylated/upregulated, hypomethylated/downregulated) were annotated and subjected to GO/KEGG analysis.

### *Phytophthora* transformation

*P. capsici* transformation was performed as previously described (51). Briefly, for overexpression, *pHAM34*-fused *PcGSTZ1* was introduced into the protoplast of LT1534 or JA8 by pTOR (GenBank: EU257520.1), while *PcGSTZ1* promoter region, instead of *pHAM34,* was tandem with *GFP* and expressed in JA8 and RJA1 by *pTOR*, respectively. For gene silencing, the reverse complementary sequences of *PcATFS1* were expressed in JA8, *PcDAMT1* and *PcDAMT3* were expressed in RJA1 and JA8 by *pHAM34* promoter, respectively. The transformants were screened in G418 PDA plates and validated by PCR and qRT-PCR or western blot.

### Electrophoretic mobility shift assay (EMSA)

EMSA assay was adapted from previously published protocols (52). PCR products were incubated with 2 nM recombinant proteins (PcDAMT1, PcDAMT2, PcDAMT3, GFP) in a final volume of 10 µL binding buffer (10 mM Tris-borate, 40 mM KCl, 5% glycerol, 1 mM MgCl_2_, 100 µM MnCl_2_, 2 mM dithiothreitol (DTT), 100 µg/mL BSA, and 1 µg/mL sonicated salmon sperm DNA, pH 7.5) at 25°C for 30 min. Then the PCR products were analyzed by 1% agarose gel electrophoresis.

### Methylated DNA immunoprecipitate-qPCR (MeDIP-qPCR)

MeDIP-qPCR was adapted from previously published protocols (15, 26). *P. capsici* gDNA was fragmented to about 400 bp using a Bioruptor Plus sonication device. The fragment DNA (5 µg) was incubated with 2 µg anti-6mA antibody (sysy) for 12–16 h at 4°C in a final volume of 500 µL MeDIP IP buffer (10 mM sodium phosphate pH 7.0, 140 mM NaCl, 0.05% Triton X-100). Pre-blocked Dynabeads Protein G (40 µL) was added and rotated at 4°C for another 2 h. After pull-down, the beads were washed five times with 1 mL MeDIP IP buffer and digested with proteinase K in 200 µL proteinase K digestion buffer (50 mM Tris-HCl pH 8.0, 10 mM EDTA, 0.5% SDS) for 3 h at 50°C.

Methylated DNA was purified using the 700 µL volume of phenol-chloroform-isopentanol, vortexed, and centrifuged at 13,000 rpm for 5 min at room temperature. The aqueous phase was transferred into a new tube and mixed with an equal volume of ethanol to precipitate the eluted DNA.

### Chromatin accessibility by real-time PCR (ChART-qPCR)

ChART-qPCR assays were performed similar to previously published studies (53, 54). JA8 and RJA1 were cultured for 4 days to harvesting. Nuclei from cells were isolated as previously described (55) in nuclei isolation buffer. Nuclei (containing 15 µg of total DNA, as measured by UV absorbance at 260 nm after lysis of an aliquot with 1% SDS) were aliquoted into 375 µL of MNase buffer (10 mM Tris pH 7.5, 4 mM MgCl_2_, 1 mM CaCl_2_, 0.32 M sucrose) and digested with 0.5 µL micrococcal nuclease (NEB, M0247) for 2 minutes at room temperature. The reaction was stopped by addition of 250 µL stop solution (0.5 M EDTA, 2% SDS, 0.15 ng/µL pTOR::GFP plasmid) and incubated at 65°C for 15 min to completely denature all protein. DNA was purified by extraction with phenol:chloroform:isoamyl alcohol and ethanol-precipitated. The DNA pellet was resuspended in 30 µL of ddH_2_O and amplified with real-time PCR using the primers for *GSTZ1* coding region (qDIPCKupr) and primers for amplifying the coding region of actin (qactin), respectively (Table S2). To control for small variations in DNA recovery during this procedure, data were normalized to the recovery of the pTOR::GFP plasmid, as assessed by real-time PCR using primers specific for the *GFP* genes (qGFP). Chromatin states can be identified based on how accessible the DNA is to nucleases. The Ct shift between digested and undigested samples indicated the susceptibility of chromatin to nuclease digestion.

### Fungicide and ferroptosis inducer/inhibitor sensitivity assay

Technical grade SYP-14288 was provided by Shenyang Research Institute of Chemical Industry (China). Other fungicides sourced commercially were fluazinam, dimethomorph, azoxystrobin, chlorothalonil, cyazofamid, cymoxanil, metalaxyl, fluopicolide, zoxamide, and oxathiapiprolin. Ferroptosis inducer erastin and inhibitor ciclopirox olamine ointment or N-acetyl-L-cysteine (NALC) were purchased from MedChem Epress (Shanghai, China). Each chemical was accurately weighed and dissolved in dimethyl sulfoxide (DMSO) or ddH_2_O to prepare solutions for different concentrations. The sensitivity assay was determined *in vitro* using a mycelia growth assay in *P. capsici* or colony inhibition assay in *E. coli* (Abs values are calculated according to △OD_600_ = OD_600 with *E.coli*_ – OD_600 blank_, OD_600 blank_ represent the LB medium added with different concentrations of SYP-14288) as described by our previous protocol (45). The diameter of *P. capsici* grown in a fungicide-positive plate was measured perpendicularly after 5 d of incubation at 25°C. The inhibitory efficacy or median effective concentration (EC_50_) were calculated as previously described (45).

### *In vitro* transformation assay of SYP-14288 by PcGSTZ1

The *in vitro* transformation of SYP-14288 by PcGSTZ1 followed previous methods (56). Through the overexpression of the protein in *E. coli* BL21 (DE3) cells, PcGSTZ1 cDNA was subcloned into the pET28a vector. Transformed cells were grown to an OD_600_ of 0.6 in LB media, and protein expression was induced with 1 mM isopropyl β-D-1-thiogalactopyranoside (IPTG) for 16 h at 20°C. The transformed *E. coli* cells were disrupted by ultrasonication in phosphate-buffered saline buffer (pH 8.0). The lysate was transferred to a Ni-NTA column, and the Ni-NTA column was washed three times using Ni-NTA wash buffer (50 mM NaH_2_PO_4_, 300 mM NaCl, 20 mM imidazole, pH 8.0). Ni-NTA elution buffer (50 mM NaH_2_PO_4_, 300 mM NaCl, 250 mM imidazole, pH 8.0) was added, and the eluate was collected. The protein PcGSTZ1 sample was concentrated using an ultra-15 centrifugal filter unit and added to 22.5 µL PBS buffer (pH 8.0). Ten milligrams per gram of SYP-14288 was incubated with 22.5 µL of 24 µM glutathione in the final 450 µL PBS buffer (pH 6.5). Then, 50 µL recombinant PcGSTZ1 was added at 30°C in a spectrophotometer for 15 min. The mix was added to PDA broth for *P. capsici* culture and detection by LC–MS/MS.

### Characterization of ferroptosis

Five canonical characteristics of ferroptosis were detected, including cellular concentrations of iron and malondialdehyde (MDA), GPx and GST activities, and mitochondrial morphology. For the iron, MDA and GSH-oxidized glutathione (GSSG) content assay, an Iron Colorimetric Assay Kit (Elabscience, E-BC-K139-S), a Lipid Peroxidation MDA Assay Kit (Beyotime, S0131S), and a GSH and GSSG Assay Kit (Beyotime, S0053) were used according to the manufacturer protocols. GPx and GST activity assays were performed using a glutathione peroxidase (GSH-PX) assay kit (Nanjing Jiancheng Bioengineering Institute, A005-1-2) and GST activity assay kit (BC0355, Solarbio, Beijing, China) following manufacturer protocols. Morphological observations of mitochondria were conducted using a transmission electron microscope (TEM) FEI Tecnai F20 (FEI, Netherlands) as previously described (57).

### Characterization of mitochondria-related function

Respiratory rate was tested using *P. capsici* isolates grown in PDB as described before (3) using an oxygen electrode. ATP content was measured with a commercial ATP assay kit (Beyotime, Shanghai, China) according to manufacturer instructions. ATP content was calculated as nanomoles per gram of protein for mycelium of *P. capsici*. The protein concentration was determined according to the bicinchoninic acid (BCA) method. Mitochondrial membrane potential (MMP) was detected using the Mitochondria Membrane Potential Assay Kit with JC-1 (Beyotime, Shanghai, China) in the protoplasts of *P. capsici*. Protoplasts were treated with JC-1, which is extensively used to detect the MMP ΔΨm, and OD_490_ was measured to evaluate the MMP. Typical uncoupler carbonyl cyanide 3-chlorophenylhydrazone was used as the positive control, and DMSO was used as a negative control. ROS detection was performed using ROS Assay Kit (S0033S, Beyotime, Shanghai, China) following the manufacturer protocol. ROS content was also calculated as nmol/g protein, and the quantity of protein was measured as previously mentioned. The content of mitochondria was represented by the amount of mtDNA detected by the method previously mentioned (58). The relative amount was calculated by mtDNA/gDNA and further compared between isolates.

### Inheritance adaptation under fungicidal stress

*P. capsici* LT1534, RJA1, and RJA2 were assessed for adaptation on SYP-14288-positive media (50 µg/mL). Mycelial plugs (5 mm in diameter) were excised from 5-day-old PDA colonies and transferred to fresh PDA plates containing the same concentration of SYP-14288. Subcultures were conducted every 5 to 7 days. Ten transfers were performed, and the 1st, 5th, and 10th subcultures were subjected to sensitivity assays and dot blots. To relieve the fungicidal stress, the isolates were grown on PDA plates without fungicide for several days after fungicidal adaptation.

### Virulence assay

Virulence of *P. capsici* isolates was tested according to the previous protocol (4). Briefly, 10^4^/mL zoospore suspensions were inoculated to 4-week-old pepper seedlings; for detached leaves, 5 mm mycelium plugs were inoculated onto *Nicotiana benthamiana* leaves. The lesion size was measured 3–5 dpi, and the disease index was calculated.

### Statistical analysis

The data were analyzed using GraphPad Prism 8 (GraphPad Software, San Diego, CA, USA). Differences between the means of two samples were determined using a *t*-test at *P* = 0.01 or 0.05. A two-way analysis of variance (ANOVA) was performed using a Sidak test at *α* = 0.05. Phylogenetic analysis was performed in MEGA5, and the blastP was performed by ClustalW and ENDscript servers.

## RESULTS

### DNA 6mA dominates in *P. capsici* genome with three DAMTs responsible for methylation

**Fig 1:**
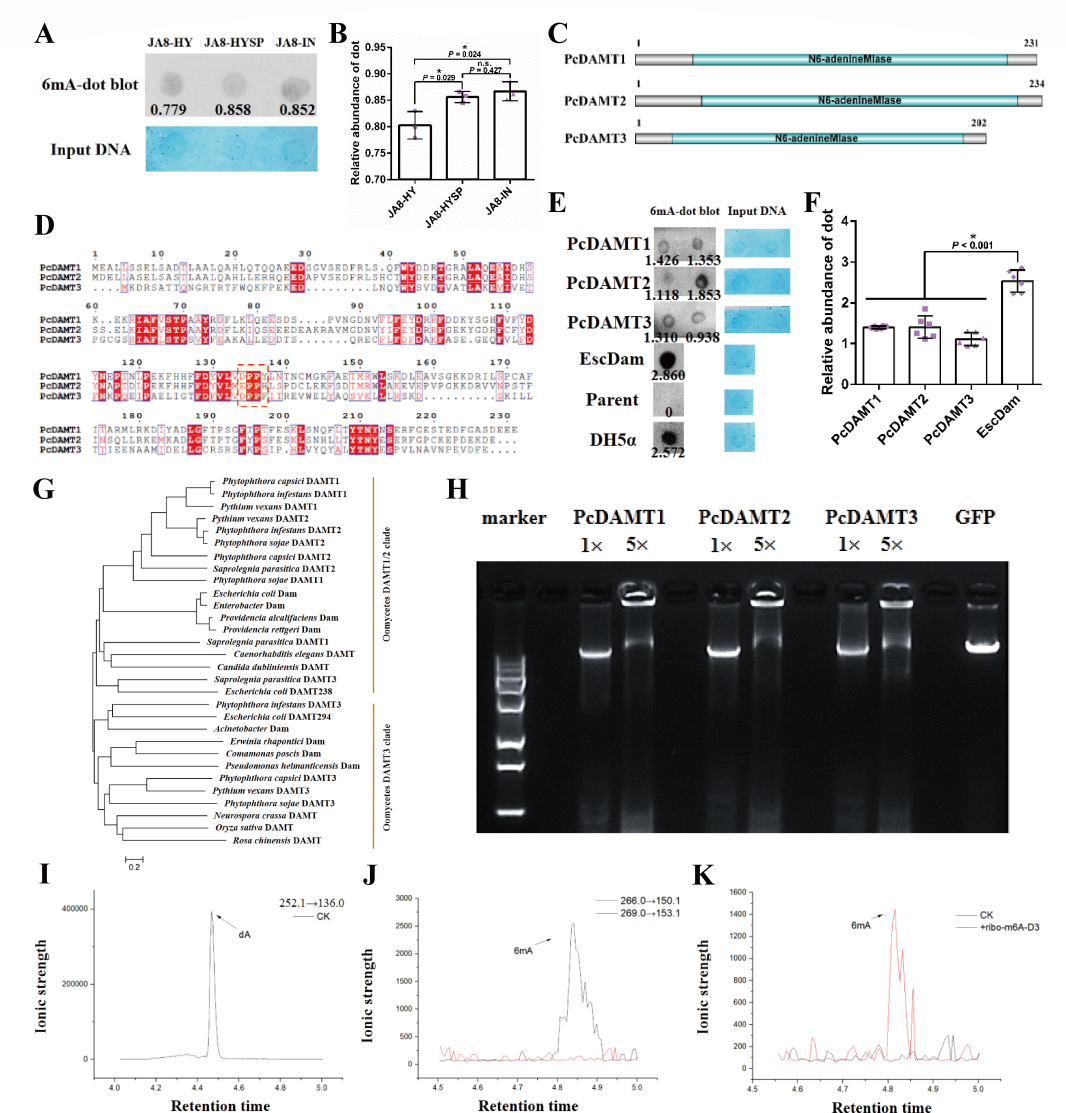
6mA occurs in *Phytophthora capsici*, and three DAMTs are responsible for DNA methylation. (**A and B**) The presence of gDNA 6mA in different life stages of *P. capsici*. HY, hyphal stage; HYSP, sporulated hypha stage; IN, infection stage. Input DNA was treated with 0.1% methylene blue solution and quantified with ImageJ. Every dot was loaded with 100 ng DNA. The experiments were independently performed in triplicate with similar results. The relative 6mA abundance was calculated (integrated signal density_6mA-dot blot_/integrated signal density_Input DNA_) with the signal quantified by ImageJ. The average 6mA abundance of each sample was represented in panel **B**. The data are represented as mean ± SD and representative of three independent experiments. (**C**) The graphic illustration of the structure of the three DAMTs in *P. capsici*. The N6-adenineMlase domain was labeled as cyan. (**D**) Sequence alignment of the three PcDAMT proteins. The conserved motifs were highlighted, and the catalytic motifs responsible for binding the methyl group of SAM were labeled with a red dashed box. (**E and F**) Bacteria methyltransferase complementation assay suggests that the three PcDAMTs have methyltransferase activity. PcDAMT1, PcDAMT2, PcDAMT3, and *Escherichia coli* Dam (EscDam) were expressed in 6mA-deficient *E. coli* isolates HST04. The DNA methylation states in the four protein-expressing strains, HST04, and the normal *E. coli* strain DH5α were detected by dot blots. The experiments were independently performed in triplicate with similar results. The average 6mA abundance in each type of strain was represented in panel **F**. The data are represented as mean ± SD and representative of six independent experiments. (**G**) Neighbor-joining tree of DAMTs and Dams in different species. (**H**) *In vitro Dpn*I-dependent DNA methylation assay indicates that the three PcDAMTs have methyltransferase activity. Recombinant proteins PcDAMT1, PcDAMT2, and PcDAMT3 were produced in TNT SP6 High-Yield Wheat Germ Protein Expression System. GFP was used as the control. The recombinant protein concentrations were 4 µg (1×) or 20 µg (5×) in each reaction. One microgram N6-methyladenine-free lambda DNA was used as the substrate, and the digestion was checked by agarose gel electrophoresis. Signal density of the *Dpn*I digestion image was analyzed using ImageJ. The experiments were performed in triplicate with similar results. (**I, J, and K**) *Phytophthora* gDNA is detected by UPLC-QqQ-MS/MS. (**I**) indicates dA (*m/z* 252.1–136.0) was detected in gDNA of *P. capsici*. (**J**) shows 6mA (*m/z* 266.0–150.1) was detected in gDNA of *P. capsici*. (**K**) indicates that RNA m6A could be transferred into gDNA. D3-m6A was added to *P. capsici* as a medium additive. gDNA of *P. capsici* was extracted, and a strong signal match D3-6mA (*m/z* 269.0–153.1) was detected. Statistical analysis by unpaired Student’s *t*-test. *P < 0.05.

Initially, whether 5mC modification is accomplished in the *P. capsici* genome was determined. As shown in Fig. S2, no 5mC immunoblot signal was detected in JA8 or LT1534. Alternatively, strong DNA 6mA signal was detected in gDNA samples of *P. capsici* by using a commercially available 6mA antibody that specifically recognizes the 6mA modification. Immunoblot signals were robustly detected in *P. capsici* samples extracted from three life stages (Fig. 1A and B). To directly confirm the presence of 6mA, hydrolyzed gDNA of *P. capsici* was analyzed by HPLC and UPLC-QqQ-MS/MS, using standard dA and 6mA as references. Peaks matching the retention time of standard dA (mass/charge ratio 252.1–136.0) and 6mA (mass/charge ratio 266.1–150.1) were present in the gDNA of *P. capsici* (Fig. 1I and J). Moreover, after introduction of D3-m6A into *P. capsici*, a strong D3-m6A (mass/charge ratio 269.0–153.1) signal in gDNA was identified, while it was absent in *P. capsici* gDNA without D3-m6A treatment (Fig. 1K). This confirmed that 6mA in gDNA could be partially transferred from m6A in RNA. Collectively, 6mA modification was shown to be naturally dominated in the *P. capsici* genome and could be partially transferred by m6A from RNA.

To identify 6mA methyltransferases, a hidden Markov model-based sequence similarity search was performed in the *P. capsici* genome. Three N6-adenineMlase domain-containing (DAMT) proteins are present in *P. capsici* (Fig. 1C). Phylogenetic analyses indicate that DAMT1/2 is conserved in all examined oomycetes and is closely related with Dam from prokaryotes, while DAMT3 is located in another clade, which is a divergent branch containing DAMTs or Dams from oomycetes, fungi, plants, and prokaryotes (Fig. 1G). The catalytic motif responsible for binding the methyl group of SAM was further examined, as shown in Fig. 1D, and DAMT1 and DAMT3 proteins have functional motifs consisting of DPPY and DPPF, respectively. However, this motif mutated into EPPH in DAMT2. This implies that the three DAMTs in *P. capsici* are involved in different pathways.

To verify the enzymatic activity of the three DAMTs, *in vitro* methyltransferase activity was tested based on 3×FLAG tagged DAMTs expressed in a cell-free protein expression system (Fig. S3). After incubation, 6mA-free lambda DNA was smeared by treatment with the *Dpn*I restriction enzyme, which recognizes the 6mA-methylated 5'-GATC-3' site in the presence of one of the three recombinant DAMTs. The methylation was heavier when more DAMTs were added, but the template DNA was intact after the control protein GFP treatment (Fig. 1H). A complementary methylation assay in a 6mA-deficient *E. coli* strain HST04 was performed, and dot blot shows that *E. coli* gDNA from DH5α, Dam-complemented HST04 transformants, and DMAT1/2/3-complemented HST04 strains had strong 6mA signals, which could not be detected in GFP-expressing strains (Fig. 1E and F). Overall, these data indicate that the three DAMTs in *P. capsici* potentially possess methyltransferase activity.

### Genome-wide DNA methylation status change is associated with SYP-14288 resistance

**Fig 2:**
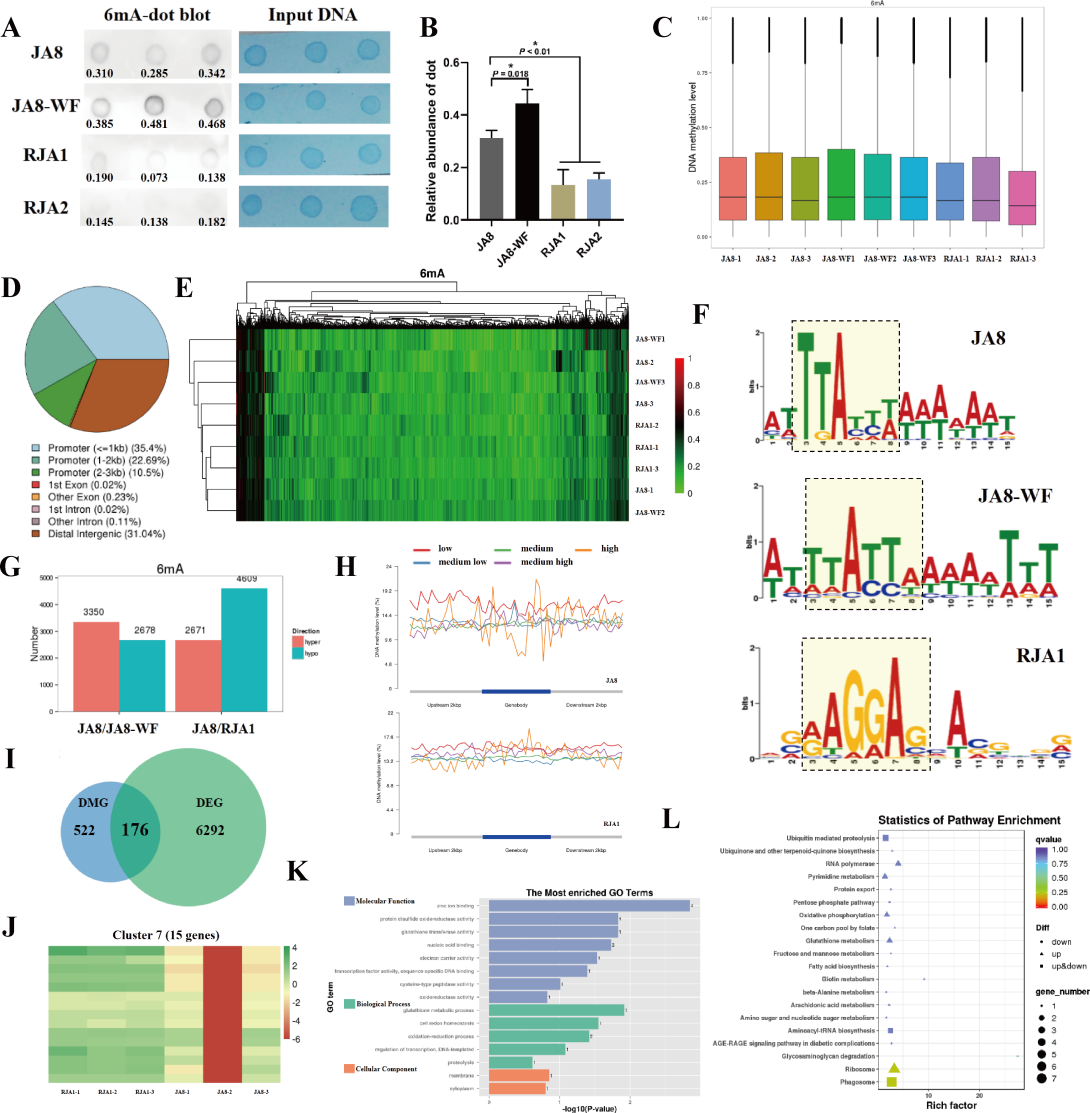
DNA 6mA and transcriptomic patterns of wild-type (JA8) and SYP-14288-resistant *Phytophthora capsici* isolates (RJA1), and the wild-type isolate under SYP-14288 treatment (JA8-WF). (**A and B**) The 6mA levels in different *P. capsici* isolates or under SYP-14288 treatment were verified by dot blot assay. RJA1/RJA2: SYP-14288-resistant isolate. Input DNA was treated with 0.1% methylene blue solution and quantified with ImageJ. Every dot loaded 100 ng DNA. The experiments were independently performed in three replicates with similar results. The relative 6mA abundance is calculated (integrated signal density_6mA-dot blot_ / integrated signal density_Input DNA_) with the signal quantified by ImageJ. The average 6mA abundance in each isolate or under SYP-14288 stress was represented in panel **B**. The data are represented as mean ± SD and representative of three independent experiments. Statistical analysis by unpaired Student’s *t*-test. *P < 0.05. (**C**) DNA 6mA level in different isolates or under SYP-14288 treatment. Three technical replicates were labeled as 1, 2, and 3. (**D**) Pie chart of 6mA distribution across *P. capsici* genome. The annotation was performed using ChIPseeker. (**E**) Clustering analysis of 6mA modified genes in JA8, JA8-WF, and RJA1. Three technical replicates were labeled as 1, 2, and 3. (**F**) Motif analysis of 6mA sites in JA8 (upper lane), JA8-WF (middle lane), and RJA1 (lower lane). The nearby 21 bp sequences of the top 1,000 6mA sites in each sample were selected and analyzed by meme algorithm and ZOOPS model. (**G**) The number of DML in JA8 vs JA8-WF and JA8 vs RJA1. (**H**) The 6mA level of differentially expressed gene in JA8 (upper lane) and RJA1 (lower lane). Genes were clustered into five categories according to their expression levels, they were defined as low (0.1 ≤ fpkm < 1), medium_low (1 ≤ fpkm < 10), medium (10 ≤ fpkm < 100), medium_high (100 ≤ fpkm < 1,000), and high (fpkm ≥ 1,000). Their 6mA levels in the upstream, downstream, or gene body regions were calculated by region-wise method. (**I**) Venn diagram illustrating the overlapping genes between DMGs and DEGs in JA8 vs RJA1 group. (**J**) Heatmap of overlapping genes of DMGs and DEGs in JA8 vs RJA1 belongs to gene group 7. (**K**) GO enrichment analysis of group 7 genes. (**L**) GO analysis of the overlapping genes of DMGs and DEGs in the JA8 vs RJA1.

The initial exposure to fungicide could induce stress responses in microbes, and then the resistance could be further developed based on the adaptive responses under fungicidal selection; therefore, the exploration of the response regulator could provide important clues to understanding the resistance mechanism (59). To illuminate the role of 6mA in SYP-14288 resistance, SYP-14288-resistant mutants RJA1 and RJA2 were generated through the domestication of a wild-type *P. capsici* isolate JA8 consistently under SYP-14288 stress. Dynamics of 6mA before and after SYP-14288 treatment as well as between RJA1, RJA2, and JA8 were explored. From dot blot analyses, the DNA 6mA levels in RJA1/RJA2 were significantly decreased compared to JA8, while it was elevated under SYP-14288 treatment in JA8 (Fig. 2A and B). This implies that 6mA modification may be an important regulator to modulate the stress response and resistance against the uncoupler in *P. capsici*. To further parse the genome-wide distribution of methylated sites, ONT-seq was performed. As shown in Fig. 2C, the methylation level was increased in JA8 after SYP-14288 treatment [JA8 treated with fungicide (JA8-WF) vs JA8 without fungicide treatment (JA8)] and decreased in RJA1 compared to JA8, in line with the results from the dot blot. The distribution of 6mA in the *P. capsici* genome is characteristic, which was reflected by dispersive DMLs (*P* < 0.05 and MethySpecificity > 0.1) that were identified in the two comparing groups (JA8 vs JA8-WF and JA8 vs RJA1, with 3,350 and 2,671 upregulated DMLs and 2,678 and 4,809 downregulated DMLs, respectively; Fig. 2G). Meanwhile, the differential methylation regions (DMRs; >10 DMLs in any ≤1 kb genomic regions) were hardly detected, reflected by 17 and 22 DMRs in JA8 vs JA8-WF group and JA8 vs RJA1 group, respectively. The numbers are significantly smaller than that in other species in which DNA methylation, like 5mC, is concentrated in certain genomic regions (60). After excluding the DMLs located far from genes (>2 kb), the gene-associated DMLs corresponded to 420 and 698 differential methylated genes (DMG) in the JA8 vs JA8-WF group and JA8 vs RJA1 group, respectively. This confirmed that the distribution of 6mA in *P. capsici* is dispersed through its genome. The distributions of 6mA loci in gene elements are shown in Fig. 2D, of which 35.4% of the modified DNA were in gene promoter regions (≤1 kb in the upstream of genes), 31.04% 6mA were in distal intergenic regions, and 0.38% 6mA were distributed inside the coding or non-coding regions of genes. Furthermore, the 6mA profiles in RJA1, JA8, and JA8-WF were verified, and the heatmap reflected a noteworthy 6mA modification change in the three types of samples (Fig. 2E). Moreover, 6mA is enriched in “TTATT(A/T)” in both JA8 and JA8-WF and is in regions with abundant adenine. The preferred motif of 6mA is converted to “(A/G)AGGAG” in RJA1 (Fig. 2F through H); this further confirms that 6mA modification is greatly altered between resistant and sensitive *P. capsici*, while it is slightly altered after SYP-14288 treatment. Altogether, these data strongly suggest that the variation of genomic 6mA modification may be a key factor in developing SYP-14288 resistance in *P. capsici* by initiating the transcription of certain genes.

The transcriptome changes between JA8 and RJA1 or JA8-WF were analyzed. Using fold change ≥2 and *P* < 0.05 as the filter criteria, 6,468 and 307 genes were identified as DEGs in JA8 vs RJA1 group and JA8 vs JA8-WF group, respectively. Slight changes in transcriptomic profiles were observed after SYP-14288 treatment, with a relative coefficient >0.926 found in all six samples of JA8-WF and JA8, which is higher than that between RJA1 and JA8 (<0.755; Fig. S4A). According to the volcano plot (Fig. S4C), 17 out of 307 DEGs were dramatically differentially expressed (fold change > 4 or < 0.25) between JA8 and JA8-WF. The 307 genes were primarily involved in endocytosis, mitogen-activated protein kinase (MAPK) signaling, protein processing in the endoplasmic reticulum, and glutathione metabolism (Fig. S4B). Therefore, 6mA modifications could be a rapid reactor that controls fungicidal stress responses in *P. capsici* through slightly modulating the expression of genes involved in xenobiotic uptake or metabolism and signal transduction. The JA8 vs RJA1 group sustained a significant transcriptome shift. Among the 6,468 DEGs, accounting for one-third of the total genes in *P. capsici*, 3,280 were upregulated and 3,188 were downregulated in RJA1 and JA8, respectively (Fig. S4D). According to DEG cluster analysis and GO enrichment, eight clusters of DEGs were classified, and the majority of the DEGs were involved in metabolic processes through catalysis or conjugation (Figure S5A and B). Accounting for 93.71% of DEGs, 6,061 genes belonged to cluster 4/5, showing no visible difference between the two isolates in the heatmap. However, 15 genes in cluster 7 were significantly upregulated in RJA1, and a xenobiotic metabolism-related gene, glutathione transferase, was included in this group (Fig. 2J and K). Considering that no xenobiotic metabolism-related pathways were enriched in other clusters (Fig. S6) and glutathione-related pathways were also involved in SYP-14288 response (Fig. S4B), the GST gene may be the key to inducing SYP-14288 resistance in *P. capsici*.

To elucidate the relationship between transcriptional changes and 6mA modifications, a combined analysis of DMRs or DMLs and DEGs was performed. The DMR-associated genes were seldom changed in RNA-seq, which further indicated that the concentrated DNA methylation in some genomic loci might be a random event without any biological functionality. Instead, diffused genomic 6mAs may induce the responses to abiotic stress. For the JA8 vs JA8-WF group, no overlapping gene existed for DMLs and DEGs, and the slight transcriptional changes could indirectly be attributed to the 6mA modifications after fungicidal treatment and further mediate stress responses against SYP-14288. Furthermore, 176 DEGs with DMLs were identified in the JA8 vs RJA1 group (Fig. 2I; File S1). The 6mA distribution in the different segments of genes with various expression levels was assessed, and the genes with the highest (fpkm ≥1,000) and lowest expression levels (0.1 ≤ fpkm < 1) showed the most fluctuant 6mA dynamics in their gene bodies and upstream/downstream sequences in JA8 (Fig. 2H); the 6mA modifications remained stable in all RJA1 genes (Fig. 2H). To further parse the pivotal genes resulting in SYP-14288 resistance through gain-of-function study, five genes (except *GST*) extracted from File S1 and were known to be xenobiotics metabolism-/transportation-associated genes were tested for their roles in resistance. As shown in Fig. S7, the separate overexpression of the five genes partially confer resistance to SYP-14288 in *E. coli*; however, the resistance was significantly weaker than that induced by a *GST* gene (*PcGSTZ1*, which is also included in File S1). Then, the KEGG enrichment was performed based on the DEGs with different DMLs in JA8 vs RJA1. As shown in Fig. 2L, among the top 20 enriched pathways, glutathione metabolism is the only pathway known to be involved in xenobiotic resistance. Furthermore, all GST metabolism-associated DEGs with DMLs were analyzed (Table S1), and among them, only *PcGSTZ1* was significantly upregulated and its gene locus was differentially methylated in JA8 and RJA1. Altogether, 6mA is a key regulator of SYP-14288 response and resistance in *P. capsici*, and glutathione-associated xenobiotic metabolism, especially *PcGSTZ1*, may be vital to induce SYP-14288 resistance through upregulation mediated by discrepant 6mA modifications.

### DAMTs are crucial in the resistance formation against SYP-14288

**Fig 3:**
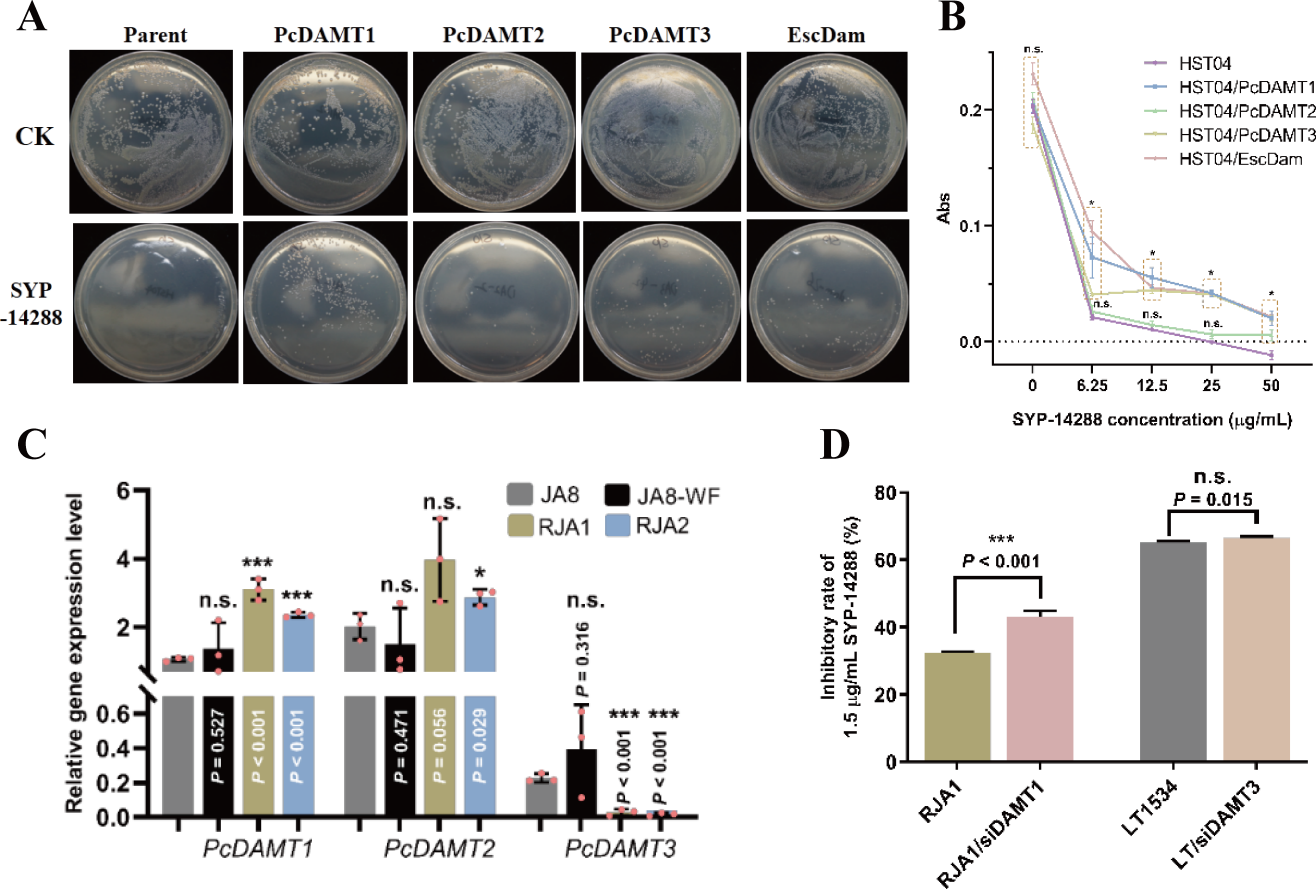
DNA adenine methyltransferases 1 is the main effector to develop SYP-14288-resistance. (**A**) The resistance against SYP-14288 was elevated in *Escherichia coli* after expressing PcDAMT1. Colonies cultured at LB medium were photographed at 24 hpi (hour post inoculation) (**B**) The minimal inhibit concentration assay was performed on different types of *E. coli* isolates. The turbidity of *E. coli* culture under different SYP-14288 concentration was measured, the Abs was calculated according to △OD_600_ = OD_600 with *E. coli*_ − OD_600 blank_. (**C**) Relative gene expression level of three *PcDAMTs* in wild-type isolate (JA8) or under SYP-14288 treatment (JA8-WF), and SYP-14288-resistant isolates (RJA1 and RJA2). *WS21* was used as internal reference. (**D**) The resistance was relieved when *PcDAMT1* was silenced in RJA1. *PcDAMT1* and *PcDAMT3* were silenced in RJA1 and JA8, respectively. The sensitivity to SYP-14288 of mutants and their parental isolates was compared at 3 dpi (day post inoculation). The data shown in B, C, and D are represented as mean ± SD and representative of at least three independent experiments. Statistical analysis by unpaired Student’s *t*-test. *P < 0.05; **P < 0.01; ***P < 0.001.

There are three DAMTs in the *P. capsici* genome, and they are responsible for DNA 6mA modifications, as previously demonstrated. To validate the hypothesis that DAMTs are involved in the resistance process against SYP-14288, the sensitivity of 6mA-deficient *E. coli* strain HST04 and three *P. capsici DAMTs*/*E. coli Dam*-complemented HST04 (HST-PcDAMT1/PcDAMT2/PcDAMT3/EscDam; Fig. S8) strains were tested against SYP-14288. As shown in Fig. 3A, the inhibitory effects of 10 µg/mL SYP-14288 on *E. coli* was dramatically decreased in *PcDAMT1* complementary strains compared to HST04, while, in *PcDAMT3*/*EscDam* complementary strains, the sensitivity against SYP-14288 was slightly decreased. This was reflected by more colonies in the three protein-expressing strains after fungicide treatment. Meanwhile, the *PcDAMT2*-expressing strain remained sensitive to the certain concentration of SYP-14288 as did HST04. The minimal inhibit concentration (MIC) assay revealed enhanced resistance to all concentrations of SYP-14288 when PcDAMT1, PcDAMT3, and EscDam were individually expressed in 6mA-deficient *E. coli*. Furthermore, above 10 ng/ml of SYP-14288, PcDAMT2/HST04 and HST04 strains showed a complete growth cessation (Fig. 3B). These data confirm that 6mA modification is important for the resistance against SYP-14288 in *E. coli*, and PcDAMT1 appears to be the core effector of resistance.

To explore the relationship between DAMTs and SYP-14288 resistance, the expression levels of three *DAMT* genes were detected. There was an increase of 2.22- to 2.92-fold for *DAMT1* and a reduction of >87% for *DAMT3* in RJA1/RJA2 compared to JA8, and the expression levels of *DAMT2* in the three isolates (fold change = 1.42–1.96) was slightly changed. The three *DAMT*s exhibit stable expression before and after SYP-14288 treatment (Fig. 3C). Furthermore, *PcDAMT1*-silencing mutants in RJA1 (RJA1/siDAMT1) and *PcDAMT3*-silencing mutants in LT1534 (LT/siDAMT3) were developed to assess whether the two differentially expressed *DAMT*s were involved in SYP-14288 resistance *in vivo*. As shown in Fig. 3D and Fig. S8, with a silence efficiency of ~40% for *PcDAMT1*, RJA1/siDAMT1 grew weaker than the parental isolate RJA1 in 1.5 µg/mL SYP-14288-amended PDA plates. The inhibitory rate increased from 32.40% (LT1534) to 43.12% (RJA1/siDAMT1). Even though the resistance could be relieved after the overexpression inhibition of *PcDAMT1* in RJA1, the silencing of *PcDAMT3* in wild-type *P. capsici*, to simulate the expression change in RJA1, resulted no change in SYP-14288 resistance (Fig. 3D and Fig. S9). These data strongly indicate that elevated expression of a terminal 6mA writer, *PcDAMT1*, is primarily responsible for SYP-14288 resistance in both *P. capsici* and prokaryotes.

### Constitutive overexpression of *PcGSTZ1* is caused by the PcDAMT1-mediated hypermethylation of its promoter region and its higher chromatin accessibility in resistant isolate

**Fig 4:**
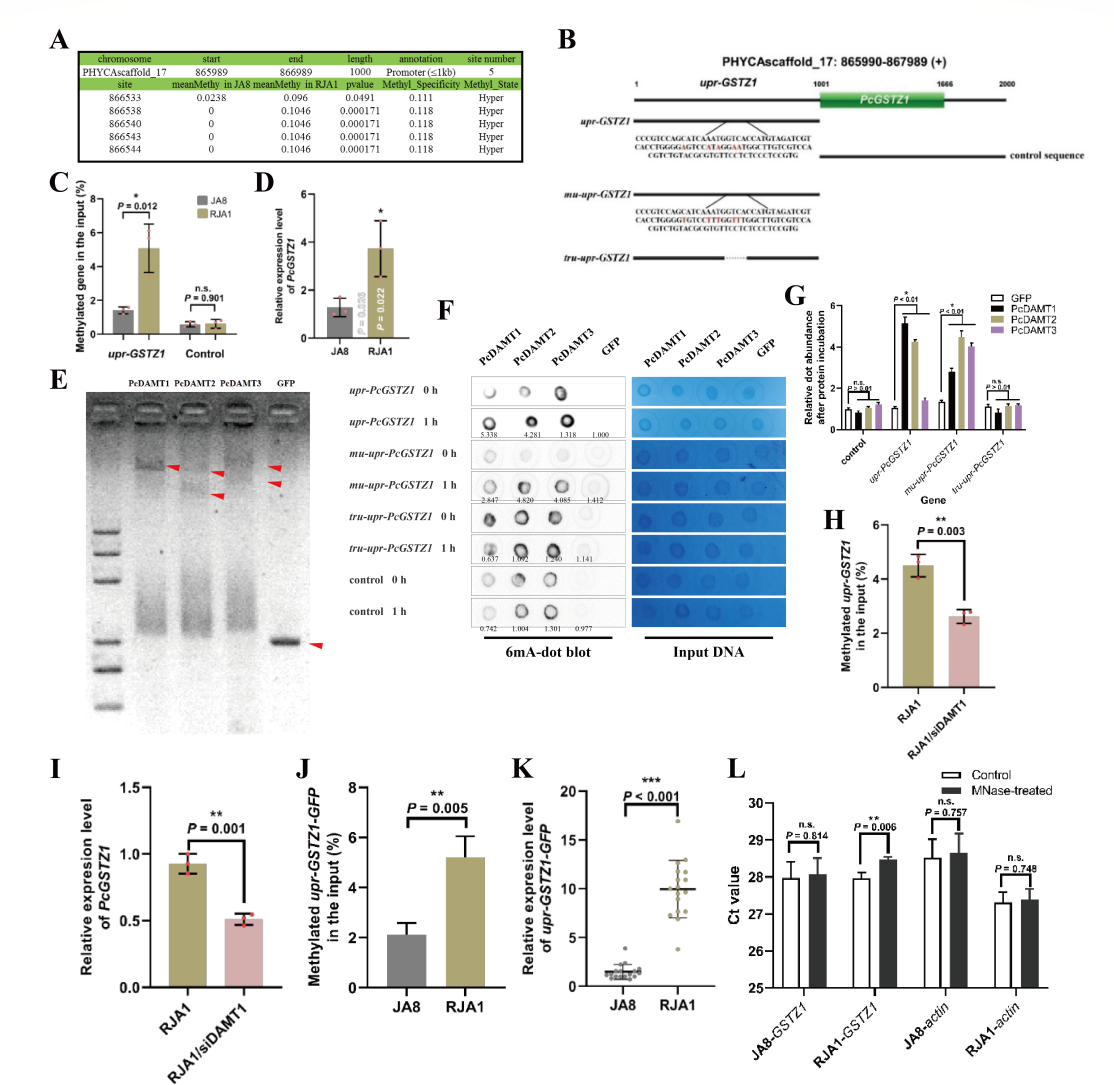
Overexpression of *PcGSTZ1* is controlled by hypermethylated 1 kb upstream region (*upr-GSTZ1*). (**A**) The five predicted 6mA sites in *upr-GSTZ1* detected by ONT-seq. (**B**) Schematic illustration of the 1 kb upstream promoter region of *PcGSTZ1* (*upr-GSTZ1*) and its mutated sequences. The downstream 1 kb sequence of *upr-GSTZ1* containing the 666 bp coding sequence of *PcGSTZ1* was used as a control. (**C**) *upr-GSTZ1* is heavily methylated in RJA1 compared to JA8. 6mA-DIP-qPCR was performed to evaluate the methylation state of *upr-GSTZ1* in two isolates. A 1 kb gene body sequence located next to *upr-GSTZ1* was used as a control. (**D**) Relative gene expression level of *PcGSTZ1* in wild-type isolate (JA8) and SYP-14288-resistant isolates (RJA1). *WS21* was used as internal reference. (**E**) EMSA indicated that *upr-GSTZ1* could efficiently be bonded by three PcDAMTs. GFP was used as a control protein. (**F and G**) *upr-GSTZ1* and *mu-upr-GSTZ1* (with five predicted methylation sites mutated version of *upr-GSTZ1*) could both be methylated by PcDAMTs but not the control protein GFP. The activity was measured by comparing the substrate methylation levels before and after incubation with each protein, which is shown by the numbers labeled below the dots (relative dot intensity of each sample at 1 h compared to the intensity at 0 h). Whole sequence substitution (control; a 1 kb gene body sequence located next to *upr-GSTZ1*) and truncated *upr-GSTZ1* (*tru-upr-GSTZ1*; the same as *upr-GSTZ1,* only missing the 100 bp sequence which contain the five predicted methylated adenines region) were used as control substrate to explore the specificity of methylation. Input DNA was treated with 0.1% methylene blue solution. The relative 6mA abundance is calculated by comparing each sample’s dot blot intensity with input DNA intensity, the intensity was quantified by ImageJ. The average 6mA abundance of each gene treated by different proteins was represented in panel **G**. The data are represented as mean ± SD and representative of three independent experiments. Statistical analysis by unpaired Student’s *t*-test. *P < 0.01. (**H**) *upr-GSTZ1* is less methylated when PcDAMT1 was silenced in RJA1. 6mA-DIP-qPCR was performed to evaluate the methylation state of *upr-GSTZ1*. (**I**) Expression level of *PcGSTZ1* was downregulated when PcDAMT1 was silenced in RJA1. *WS21* was used as internal reference. (**J and K**) *upr-GSTZ1* could trigger a stronger expression of its tandem gene in RJA1 than JA8 by hypermethylation. Transformation of *upr-GSTZ1-GFP* in RJA1 or JA8 background could result in significant gene expression difference (**J**) and methylation difference (**K**) detected by qRT-PCR and 6mA-DIP-PCR, respectively. (**L**) Chromatin accessibility of *upr-GSTZ1*. Chromatin accessibility was determined according to the susceptibility to MNase. Ct value of *upr-GSTZ1* and a control gene, the promoter region of *actin*, were detected by qPCR and compared between control and MNase-treated samples. The significant change in *upr-GSTZ1* of RJA1 indicated that chromatin is more open in the isolate. The data shown in panels C, D, and H-L are the mean ± SD and representative of at least three independent experiments. All the experiments were performed in triplicate with similar results. In panels C, D, H to L, statistical analysis by unpaired Student’s *t*-test. *P < 0.05; **P < 0.01; ***P < 0.001.

According to ONT-seq, the 1 kb upstream region of *PcGSTZ1* (*upr-GSTZ1*) is hypermethylated in RJA1 compared to JA8 (Fig. 4A), which may result in gene overexpression. To confirm the methylation state in *upr-GSTZ1*, 6mA-DIP-qPCR was performed. Compared to the control sequence (1 kb gene body sequence located next to *upr-GSTZ1*; Fig. 4B) that was weakly bonded by 6mA antibody with no difference in 6mA abundance between RJA1 and JA8, the *upr-GSTZ1* was 3.60-fold enriched in RJA1 than in JA8 after 6mA antibody incubation (Fig. 4C). This indicates that the *upr-GSTZ1* is highly but specifically methylated in RJA1 compared to JA8. To determine whether hypermethylation in *upr-GSTZ1* could promote the expression of *PcGSTZ1* in RJA1, as shown in RNA-seq, qRT-PCR was conducted to confirm gene expression. From Fig. 4D, *PcGSTZ1* expression was significantly elevated (2.924-fold) in RJA1 than in JA8. This proves that hypermethylation of *upr-GSTZ*1 could activate *PcGSTZ1* expression in *P. capsici*. To confirm the interaction between *upr-GSTZ1* and DAMTs, an EMSA was performed. As shown in Fig. 4E, the *upr-GSTZ1* fragment could be strongly bonded by three PcDAMTs but not the GFP control protein. Additionally, an *in vitro* methylation assay confirmed that *upr-GSTZ1* is specifically methylated by the three DAMTs in *P. capsici* (Fig. 4F and G). Interestingly, the mutation of the five predicted methylated adenines (mu-upr-GSTZ1) did not affect the methylation on *upr-GSTZ1* (Fig. 4B and F). Nevertheless, only whole sequence substitution (control; a 1 kb gene body sequence located next to *upr-GSTZ1*) and truncated *upr-GSTZ1* (tru-upr-GSTZ1; the same with *upr-GSTZ1,* only missing the 100 bp sequence which contain the five predicted methylated adenines region) were no longer methylated after incubation with PcDAMTs (Fig. 4B and F). These results indicate that the core 100 bp sequence of *upr-GSTZ1*, but not the five predicted methylation sites, could be efficiently bonded and methylated by all three PcDAMTs *in vitro*; the *in vivo* effector of *upr-GSTZ1* methylation is further studied.

As the major effector of DNA methylation in *P. capsici* and upregulated with the hypermethylation of *PcGSTZ1* in RJA1 compared with JA8, PcDAMT1 was speculated as the key factor triggering the hypermethylation in *PcGSTZ1*. The *upr-GSTZ1* methylation state and *PcGSTZ1* expression level in RJA1/siDAMT1 and RJA1 were compared, and comparison confirmed that, with a 41.87% reduction of methylation in *urp-GSTZ1* region, RJA1/siDAMT1 displayed an 44.89% decrement in *PcGSTZ1* expression compared to RJA1 (Fig. 4H and I). To confirm the *in vivo* influence of DNA methylation state on *upr-GSTZ1* tandem gene expression, the methylation state of *upr-GSTZ1* fused *GFP* gene (*upr-GSTZ1-GFP*) in RJA1 and JA8 was assessed, and a markedly different 6mA methylation level in the two isolates was observed (a 2.46-fold increase of the *upr-GSTZ1-GFP* 6mA level in RJA1 than in JA8; Fig. 4J). Furthermore, the heavily methylated promoter resulted in a higher expression level of *GFP* (mean relative abundance of 9.96 in RJA1 and 1.50 in JA8; Fig. 4K). In total, PcDAMT1 is the key to modulating the expression of *PcGSTZ1* through hypermethylation at its *upr-GSTZ1* region.

To further explain the relationship between *PcGSTZ1* overexpression and hypermethylation, chromatin accessibility assay was performed. A significant elevation in chromatin accessibility was found in RJA1 compared to JA8 (Ct value of *upr-GSTZ1* increased by 0.09 and 0.50 in JA8 and RJA1 after MNase treatment, respectively; Fig. 4L), which indicated that the gene locus of *upr-GSTZ1* was more susceptible to MNase digestion in RJA1. As a control gene, the Ct value of the promoter region of *actin* in the two isolates both remained stable before or after MNase treated (Fig. 4L); this implied that the elevation in chromatin accessibility specifically happened in the methylated genomic loci. Therefore, the higher chromatin accessibility in RJA1 could provide more contact possibilities to transcription factors (TFs), which may directly induce the overexpression of *PcGSTZ1*.

### PcGSTZ1 is responsible for the development of SYP-14288 resistance through fungicide metabolism

**Fig 5:**
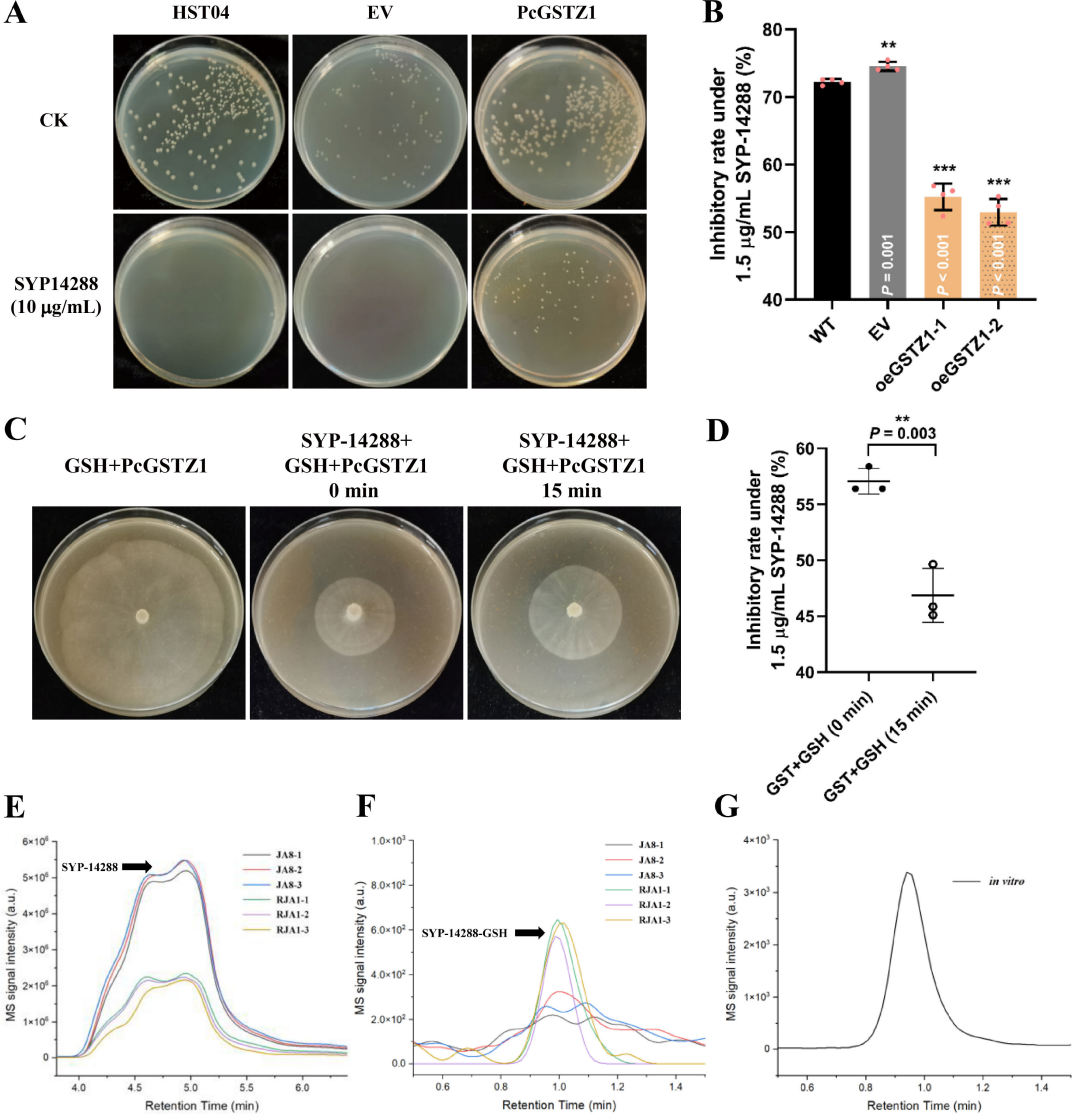
PcGSTZ1 is responsible for SYP-14288 resistance by its detoxification. (**A**) The resistance against SYP-14288 (10 µg/mL) was elevated in *Escherichia coli* after expressing PcGSTZ1. Colonies cultured in LB medium were photographed at 24 hpi (hour post inoculation). (**B**) The resistance against SYP-14288 was elevated in *Phytophthora capsici* when PcGSTZ1 was overexpressed. The inhibitory effect of SYP-14288 on wild-type (JA8), empty vector (EV), and two PcGSTZ1 overexpression isolates (oeGSTZ1-1 and oeGSTZ1-2) was detected at 3 dpi. (**C and D**) PcGSTZ1 could efficiently detoxify SYP-14288. PcGSTZ1 protein was produced in *E. coli*. Ternary system containing SYP-14288, GSH, and PcGSTZ1 were incubated for 0 and 15 min, respectively. The sensitivity of *P. capsici* against the outcome product was tested. Mycelial colonies were photographed at 3 dpi (**C**). The inhibitory effect of the products produced from the system after incubating for 0 or 15 min on *P. capsici* was detected at 3 dpi (**D**). (**E**) SYP-14288 was significantly decreased in RJA1 compared to JA8. 1.5 µg/mL SYP-14288 was added to liquid PDA medium, and *P. capsici* JA8 and RJA1 were incubated in the medium for 24 h. The amount of SYP-14288 in the two isolates was detected by HPLC. (**F**) SYP-14288-GSH was detected in RJA1 but not in JA8. 1.5 µg/mL SYP-14288 was added to liquid PDA medium, and JA8 and RJA1 were incubated in the medium for 24 h. SYP-14288-GSH was detected by HPLC. (**G**) The retention time of SYP-14288-GSH in HPLC. *In vitro* incubation of SYP-14288, GSH, and PcGSTZ1 for 15 min; the resulting product, SYP-14288-GSH, was detected by HPLC. The data shown in B and D are represented as mean ± SD and representative of three independent experiments. Statistical analysis by unpaired Student’s *t*-test. *P < 0.05; **P < 0.01; ***P < 0.001.

To explain the relationship of PcGSTZ1 with SYP-14288 resistance, *PcGSTZ1* was overexpressed in *E. coli* and *P. capsici* (Fig. S10). As shown in Fig. 5A and B, the elevation of *PcGSTZ1* enhanced fungicidal resistance in both *E. coli* and *P. capsici*. For *E. coli*, HST04 and empty vector-harboring strains could not be grown on LB amended with 10 µg/mL SYP-14288, while several colonies of *PcGSTZ1*-expressing strain emerged whether or not fungicide was present. Moreover, the inhibitory efficiencies dropped sharply from 72.24% to 52.94%−55.21% in *P. capsici* compared to the parental isolate under 1.5 µg/mL SYP-14288 treatment.

GSTZ1 is well known for its function in xenobiotic metabolism, and this study tested if it could catalyze the detoxification of SYP-14288. Using HPLC-MS, a chelate compound, generated after the *in vitr*o co-incubation of GSH and SYP-14288 in the presence of PcGSTZ1, was identified and was subsequently referred to as GSH-SYP (retention time = 0.95 min, m/z = 725.0; Fig. 5G and Fig. S11). The amount of SYP-14288 (retention time = 4.8 min, m/z = 418.9) was significantly decreased, but the GSH-SYP was markedly increased in RJA1 compared to JA8 (Fig. 5E and F). Furthermore, the bioactivity of GSH-SYP exhibited a significantly weaker inhibitory activity than the parent compound on *P. capsici* (46.87% of GSH-SYP and 57.06% of SYP-14288 at the same dosage of SYP-14288; Fig. 5C and D). Altogether, the data show that PcGSTZ1 can efficiently catalyze the transformation of SYP-14288 to GSH-SYP, which is weaker than SYP-14288 in inhibitory effect. Overexpression of *PcGSTZ1* can lead to resistance through rapidly reducing the toxicity of SYP-14288 in target organisms.

### SYP-14288 resistance advances ferroptosis and fitness penalty by triggering ROS burst and mitochondrial damages

**Fig 6:**
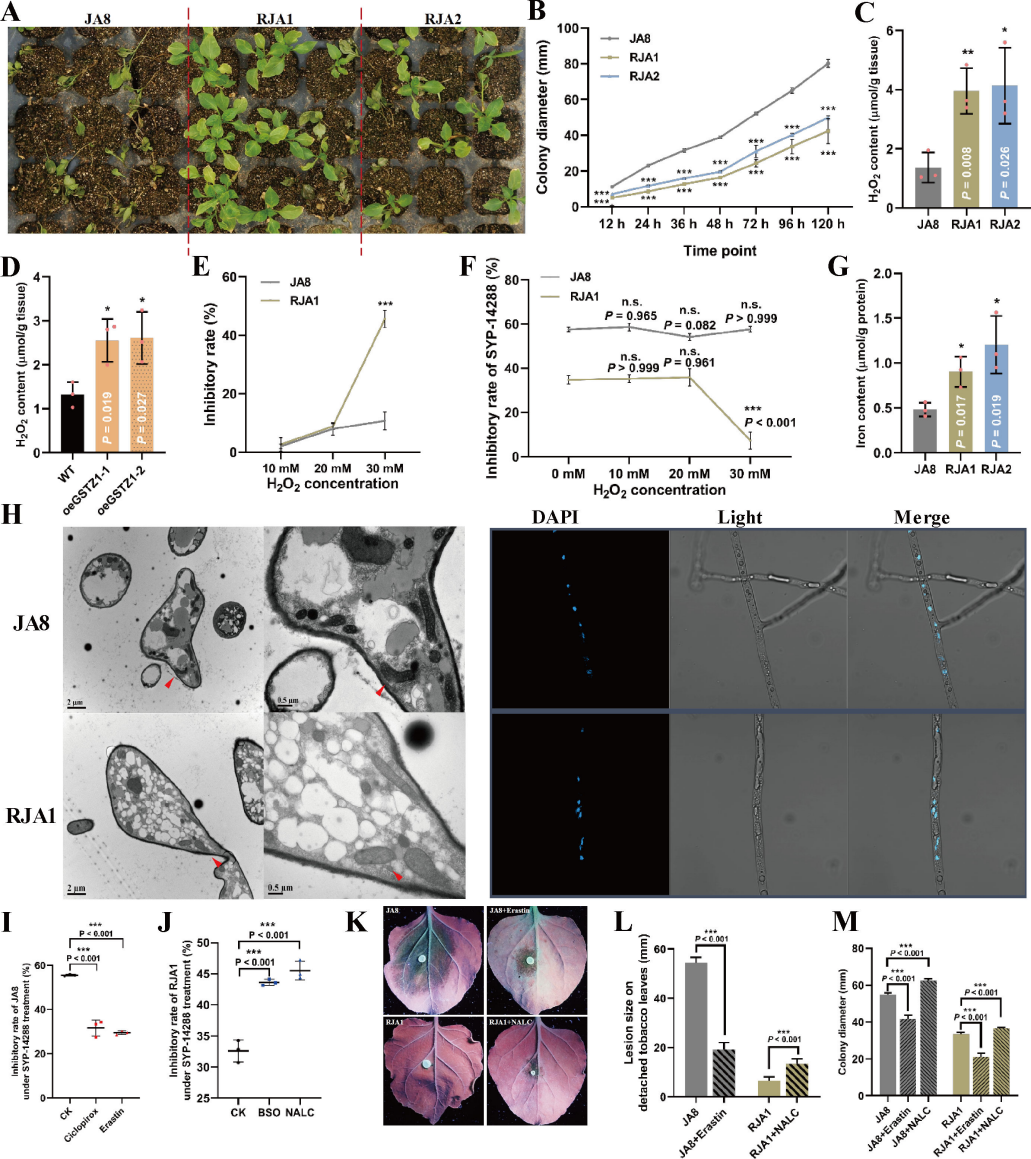
Resistance against SYP-14288 confers ferroptosis and fitness penalty in *Phytophthora capsici*. (**A**) Virulence of SYP-14288-resistant isolates was significantly reduced compared to the parental isolate. Equal zoospore suspensions (10^4^ /mL) of wild-type JA8 and SYP-14288-resistant isolates (RJA1 and RJA2) were inoculated on chili pepper (4 weeks old), infected seedlings, and photographed at 4 dpi (day post inoculation). (**B**) Hyphal growth rate was decreased in SYP-14288-resistant isolates RJA1/RJA2 compared to the parental isolate JA8. The colony diameters of the three isolates at different time points were measured, and the data at the same time point were compared. The slope of the growth curve represents the growth rate. (**C and D**) Cellular H_2_O_2_ content was increased in SYP-14288-resistant isolates (**C**) and *PcGSTZ1* overexpression mutants (**D**) compared to parental isolates JA8 or LT1534, respectively. H_2_O_2_ content in the hypha of different isolates was detected by commercial kit, and the content was adjusted by the weight of tested tissues. (**E**) RJA1 was hypersensitive to high concentrations of H_2_O_2_. JA8 and RJA1 were inoculated onto PDA medium containing different concentrations of H_2_O_2_; empty PDA medium was used as control. The sensitivity of *P. capsici* to H_2_O_2_ was reflected by the inhibition of hyphal growth. Data at the same H_2_O_2_ concentrations were compared. (**F**) The inhibitory effect of SYP-14288 on RJA1 was relieved in the presence of high H_2_O_2_ concentration. Growth states of JA8 and RJA1 on H_2_O_2_-only plates and plates containing both H_2_O_2_ and SYP-14288 were detected. The inhibition induced by SYP-14288 = (inhibitory rate_H2O2_ − inhibitory effect_H2O2+SYP-14288_ at the same H_2_O_2_ concentration) / inhibitory rate_H2O2_. Data at the different H_2_O_2_ concentrations in single isolates were compared. (**G**) Iron content, the canonical character of ferroptosis, was significantly elevated in SYP-14288-resistant isolates compared to the parent. The content was adjusted by per gram of protein extracted from tissues. (**H**) Mitochondrial phenotype (left panel) and nucleus morphology (right panel) of JA8 and RJA1. Mitochondria observation was performed by TEM. Nucleus observation was performed by confocal microscopy after 4′,6-diamidino-2-phenylindole (DAPI) treatment. (**I**) Sensitivity against SYP-14288 of JA8 was reduced after triggering ferroptosis. Two ferroptosis inducers were combined with SYP-14288. The inhibitory effects of fungicide-only and fungicide + ferroptosis inducer on JA8 were detected. (**J**) Resistance against SYP-14288 was relieved when ferroptosis was inhibited in RJA1. (**K and L**) Fitness penalty (virulence) was induced by ferroptosis inducer in JA8 and reversed by ferroptosis inhibitor in RJA1, respectively. Eight-week-old *Nicotiana benthamiana* leaves were inoculated with JA8 or RJA1 grown on PDA medium or ferroptosis inducer/inhibitor-added PDA medium. Five-millimeter hyphal plugs were inoculated onto each leaf, the lesion size was measured, and the infected leaves were photographed both at 3 dpi. (**M**) Fitness penalty (hyphal growth) was induced by ferroptosis inducer and reversed by ferroptosis inhibitor both in JA8 and RJA1. All the data shown in Fig. 6 were representative of at least three independent experiments. The data are as mean ± SD. Data in panels B to E, G, S, and I to M were statistically analyzed by unpaired Student’s *t*-test. Data in panel F were statistically analyzed by two-way ANOVA. *P < 0.05; **P < 0.01; ***P < 0.001.

Fitness penalty is commonly observed in MFR pathogens (8). Herein, the growth vigor of SYP-14288-resistant isolates RJA1/RJA2 is much weaker than that in JA8, which is reflected by weaker virulence and slower growth rate (Fig. 6A and B). Since *GSTZ1* overexpression can induce ROS burst and causes ferroptosis in hepatocellular carcinoma cells (35), this investigation aimed to determine if ferroptosis and fitness penalty are triggered by GSTZ1-mediated resistance. As expected, H_2_O_2_ concentrations in RJA1/RJA2 and oeGSTZ1 were significantly higher than that in JA8 and LT1534, respectively (>2.90-fold increase in RJA1/RJA2 compared to JA8, >1.93-fold increase in oeGSTZ1 compared to LT1534; Fig. 6C and D). Therefore, *PcGSTZ1* overexpression, triggered by 6mA modification, could efficiently increase the cellular ROS content. ROS burst is likely induced by mitochondrial damage in RJA1. Two major parameters of mitochondrial function (26), oxygen consumption level and ATP content, were increased and reduced in SYP-14288-resistant isolates compared to JA8, respectively (Fig. S13H and I). Furthermore, RJA1 was more sensitive to exogenous ROS than JA8 (Fig. 6E); this was possibly due to the intolerance caused by the inherently high amounts of ROS in RJA1. Surprisingly, compared to H_2_O_2_-only treatment, combined applications of exogenous H_2_O_2_ at high concentrations and SYP-14288 could relieve the inhibitory effect from SYP-14288 on RJA1, but not JA8 (Fig. 6F). This implies that ROS-related cellular affairs may be triggered more easily in RJA1 and, thus, counteract the inhibitory effects of SYP-14288. Like the findings in *C. elegans* that the ATFS1 could modulate 6mA modifiers and facilitate the activation of mitochondrial stress response genes (26), it was revealed here through a hidden Markov model that a bZIP transcriptional factor with mitochondrial localization signal (MTS) (mitochondrial localization was predicted by MitoProt II, MTS score = 0.6054, which is similar to the canonical MTS with the value 0.8602 and significantly higher than any other bZIP proteins in *P. capsici*; File S2), PcATFS1, was significantly downregulated in SYP-14288-resistant isolates compared with JA8 (Fig. S12A). Its expression level was decreased after exogenous ROS treatment (Fig. S12B). Interestingly, the gene expression levels of *PcDAMT1*, *PcDAMT2,* and *PcGSTZ1* were all dramatically elevated when *PcATFS1* was silenced, while the expression of *PcDAMT3* was repressed in siPcATFS1 compared with the parental isolate (Fig. S12C). These changes were similar with that in RJA1/2 compared with JA8. Thus, mitochondrial impairment-induced ROS burst in SYP-14288-resistant isolates resulted in hypersensitivity to H_2_O_2_, which then affected the *PcATFS1* expression and further modulated the 6mA profile in *P. capsici* by regulating the expression of *PcDAMT*s. Furthermore, whether cellular events like ferroptosis caused by high concentrations of H_2_O_2_ were involved in the resistance elevation against SYP-14288 in *P. capsici* was explored.

To verify if ferroptosis happened in SYP-14288-resistant isolate and the relationship between *PcGSTZ1* overexpression and ferroptosis, canonical characteristics of ferroptosis were examined in RJA1 and oeGSTZ1. As shown in Fig. 6G and S13A–C, iron and MDA contents were significantly increased in SYP-14288-resistant isolates and oeGSTZ1. GSSG and GSH concentrations in SYP-14288-resistant isolates were significantly reduced than in JA8 isolates (Fig. S13F and G). The GST activity was enhanced by 1.71- to 2.01-fold, and GPxs were decreased by 20.56%–30.54% in RJA1/RJA2 compared to JA8, respectively (Fig. S13D and E). GPxs catalyze the reduction of H_2_O_2_ or organic hydroperoxides to water or corresponding alcohols using reduced GSH. Therefore, the impairment of GPx activity could enhance ROS burst in SYP-14288-resistant isolates (61). Despite GPx4, the core regulator of ferroptosis, being absent in microbes (62), a GPx with variably methylated promoter, *PcGPx1* (upregulated for 3.51- to 5.62-fold in resistant isolates; Fig. S14), was further studied as a representative GPx to examine their role in uncoupler resistance. Interestingly, with the overexpression of *PcGPx1*, the sensitivity against SYP-14288 increased compared to LT1534 (Fig. S14), which implies that the disturbance of ROS metabolism could enhance the effect of uncouplers. Altogether, these data confirm that ROS burst and ferroptosis could participate in uncoupler resistance. Meanwhile, mitochondria in RJA1 were swollen and inflated (Fig. 6H), and the total number of mitochondria increased in RJA1 (Fig. S13J). Moreover, the MMP was dramatically reduced in SYP-14288-resistant isolates compared to JA8 (Fig. S13K), which further indicates that the membrane integrity and function of mitochondrion were damaged after resistance developed. With the nuclear morphology in RJA1 and JA8 remaining the same (Fig. 6H), all parameters mentioned above demonstrate that ferroptosis occurred in SYP-14288-resistant isolates (Fig. S13L). Furthermore, the ferroptosis inducer ciclopirox or erastin and inhibitor buthionine sulfoximine (BSO) or NALC was introduced to confirm whether ferroptosis could be involved in SYP-14288 resistance. As expected, ciclopirox or erastin could efficiently reduce the sensitivity against SYP-14288 in JA8 (inhibitory rate dropped from 55.50% to 31.71% or 29.48% under SYP-14288 combined with ciclopirox or erastin treatment, respectively; Fig. 6I), and it repressed ferroptosis in RJA1, leading to a partial loss of resistance (inhibitory rate raised from 32.60% to 43.61% or 45.51% under SYP-14288 combined with BSO or NALC treatment, respectively; Fig. 6J). Interestingly, JA8 infection dropped when ferroptosis was stimulated, and the lost virulence of RJA1 was partially recovered after ferroptosis inhibition (Fig. 6K and L). Meanwhile, hyphal growth rates were decreased and increased in JA8 and RJA1 after ferroptosis was induced or inhibited, respectively (Fig. 6M). This indicates that *GSTZ1* overexpression-induced ferroptosis is involved in fitness penalty present in uncoupler resistance. This new evidence shows that ferroptosis and mitochondrial damage caused by ROS accumulation could result in the impaired growth vigor of SYP-14288-resistant isolates, which ultimately effects the living costs and uncoupler resistance.

### The 6mA-GSTZ1-ferroptosis axis is responsible for MFR, and lower genomic 6mA level enables effective intergenerational inheritance of stress adaptation in *P. capsici*

**Fig 7:**
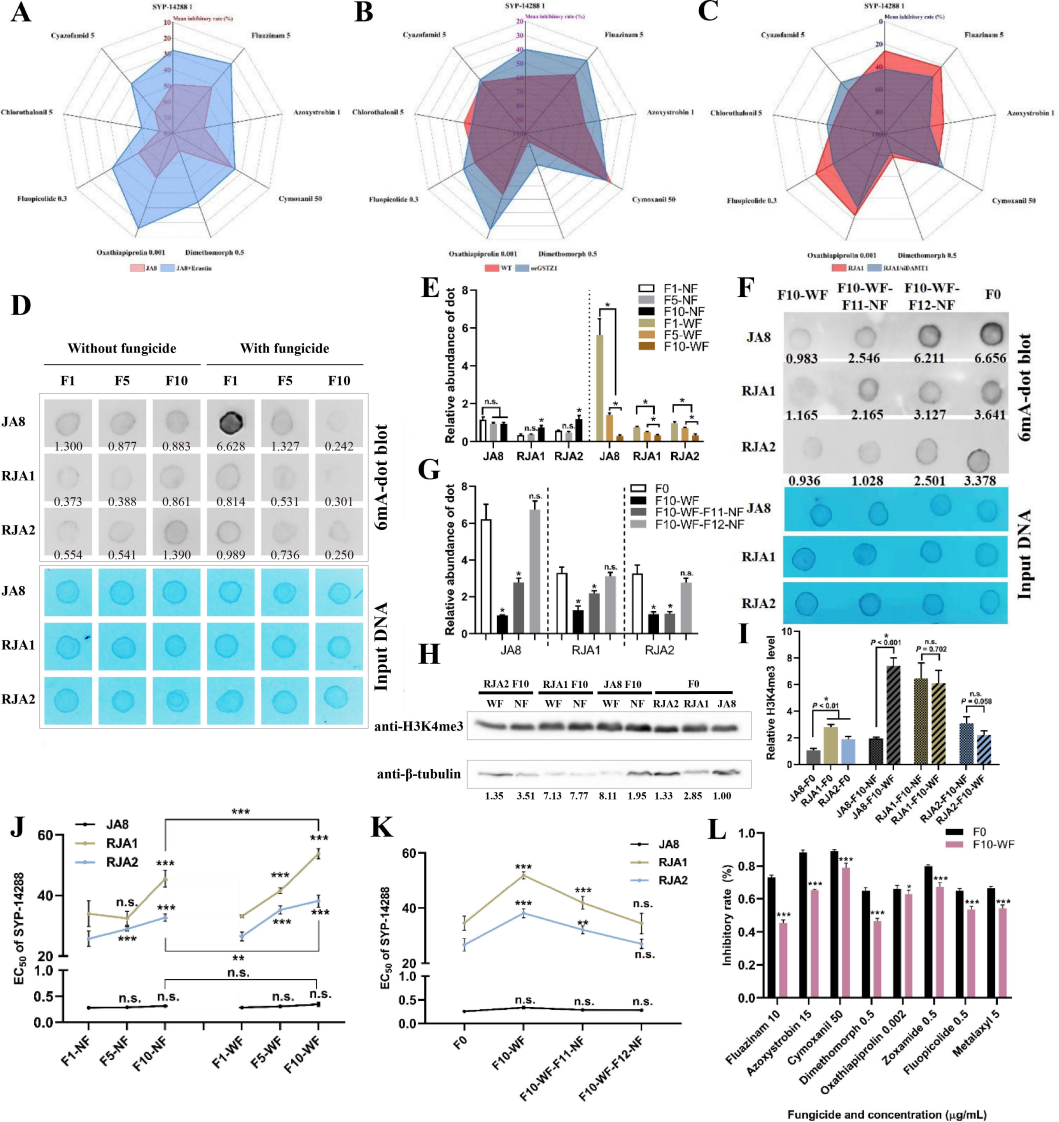
The 6mA-GSTZ1-ferroptosis regulatory axis is involved in transgenerational inheritance of multi-fungicides resistance and promoting quick adaptation under fungicidal stress in *Phytophthora capsici*. (**A, B, and C**) Sensitivity of different isolates or under ferroptosis inducer treatment against various fungicides. (**A**) Wild-type *P. capsici* JA8 before and after ferroptosis inducer treatment. (**B**) Wild-type *P. capsici* LT1534 and *PcGSTZ1* overexpression mutant oeGSTZ1 generated in LT1534 background. (**C**) SYP-14288-resistant isolate RJA1 and *PcDAMT1*-silenced mutant RJA1/siDAMT1 generated in RJA1 background. The inhibitory rate was numbered. The concentration of each fungicide was labeled (μg/mL). (**D, E, F, and G**) The 6mA levels in *P. capsici* isolates with (WF) or without (NF) SYP-14288 treatment for different generations were verified by dot blot assay. RJA1/RJA2: SYP-14288-resistant isolate. (**D and E**) Persistently cultured JA8, RJA1, and RJA2 on empty PDA plate or 1.5 µg/mL-SYP-14288-added PDA plate for 10 generations. DNA from the isolates after incubation for 1, 5, and 10 generations were extracted and detected. (**F and G**) The three isolates consistently cultured on SYP-14288 PDA plate for 10 generations were subsequently cultured on empty PDA plates for two generations. DNA 6mA level of the isolates after incubation for another one or two generations were compared. Input DNA was treated with 0.1% methylene blue solution and quantified with ImageJ. Every dot loaded 100 ng DNA. The experiments were independently conducted in three replicates with similar results. The relative 6mA abundance is calculated (integrated signal density_6mA-dot blot_ / integrated signal density_Input DNA_) with the signal quantified by ImageJ. In panels **E** and **G**, the average 6mA abundance of each isolate after incubation for different generations was represented as mean ± SD and representative of three independent experiments. Statistical analysis by unpaired Student’s *t*-test. *P < 0.01. (**H and I**) Histone H3K4me3 levels in JA8, RJA1 and RJA2 before and after incubation with/without fungicide for 10 generations. The average H3K4me3 abundance of each isolate or under different conditions was represented in panel **I**. The data are represented as mean ± SD and representative of three independent experiments. Statistical analysis by unpaired Student’s *t*-test. *P < 0.01. β-tubulin was used as control. (**J**) Sensitivity of JA8, RJA1, and RJA2 to SYP-14288 after incubation with or without fungicide for different generations. NF, no fungicide treatment. WF, with fungicide treatment. (**K**) Sensitivity of JA8, RJA1, and RJA2 to SYP-14288 at different states. F0, original isolates. F10-WF, incubated with SYP-14288 for 10 generations. F10-WF-F11/F12-NF, abolished fungicidal stress for one or two generations after treating with SYP-14288 for 10 generations. (**L**) Sensitivity of JA8 and JA8 treated with SYP-14288 for 10 generations to fungicides belonging to different mode of actions. The concentration of each fungicide was labeled (μg/mL). All the data shown in panels **J, K, and L** were representative of at least three independent experiments. The data shown are as mean ± SD and (J or K) statistical analysis by two-way ANOVA (Tukey test). (**L**) was statistically analyzed by unpaired Student’s *t*-test. *P < 0.05; **P < 0.01; ***P < 0.001.

Physiological changes accompanying uncoupler resistance appear to be pleiotropic in bacteria (63). SYP-14288 has cross-resistance with some fungicides in *P. capsici* (3). Herein, the sensitivities of RJA1 and JA8 were measured against almost all commercial fungicides belonging to different modes of action (MoA), divided by FRAC (https://www.frac.info/home), which target oomycete plant diseases. As shown in Table S3, the EC_50_s against fluazinam, azoxystrobin, oxathiapiprolin, zoxamide, fluopicolide, and chlorothalonil increased by 3.04- to 22.62-fold in RJA1 compared to JA8. On the contrary, RJA1 were more sensitive to cymoxanil and cyazofamid than JA8; the EC_50_s were decreased to 30% in RJA1 compared with JA8, which was likely caused by biochemical changes and downstream effects when treated with fungicides belonging to different MoAs in resistant isolates. To further explore whether the 6mA-GSTZ1-ferroptosis axis could contribute to MFR, the sensitivities of RJA1/siDAMT1, oeGSTZ1, erastin-treated *P. capsici* isolates, and their parental isolates were exposed to different fungicides. As shown in Fig. 7A, stimulation of ferroptosis strongly benefited resistance development against all tested fungicides except cymoxanil. Interestingly, overexpression of *PcGSTZ1* in LT1534 or silencing of *PcDAMT1* in RJA1 significantly enhanced or relieved the resistances against many fungicides, including uncouplers azoxystrobin, dimethomorph, oxathiapiprolin, and fluopicolide, respectively (Fig. 7B and C). The data indicate that the 6mA-GSTZ1-ferroptosis axis could also modulate the resistance against many fungicide types, including uncouplers.

Heritable changes in 6mA are associated with transgenerational inheritance of responses to mitochondrial stress in *C. elegans* (26), which enables 6mA to be an epigenetic marker that transmits adaptive advantage to progeny. Herein, whether and how the epigenetic marker could be transmitted in *P. capsici* was determined. The results showed that the 6mA level was gradually elevated in RJA1/RJA2 and remained stable in JA8 when continuously transferred for 10 generations without fungicide treatment (Fig. 7D and E). Interestingly, with persistent SYP-14288 treatment, the 6mA levels in all three isolates sharply declined (Fig. 7D and E). Meanwhile, as an accompanying epigenetic marker of 6mA (26), H3K4me3 modification increased in RJA1/RJA2 and was continuously elevated after 10 generations compared with JA8 without fungicide treatment (Fig. 7H and I). In SYP-14288-containing plates, H3K4me3 abundance was dramatically increased in JA8 after 10 successive adaptations but remain unchanged in RJA1/RJA2 (Fig. 7H and I). As H3K4me3 acts as a transcriptional activator, its elevation in SYP-14288-resistant isolates and in JA8 after successive fungicidal treatments could confer chromatin remodeling and further enhance gene expression to combat fungicidal stress. Moreover, consistent with the variation of epigenetic modifications, the resistance against SYP-14288 was further elevated in RJA1/RJA2 when persistently treated with fungicide but remained stable in JA8 regardless of SYP-14288 stress (Fig. 7J). These data indicate that the inherent lower 6mA level and higher H3K4me3 level act as dynamic epigenetic modifications among generations and enable more rapid response of stress in SYP-14288-resistant isolates. The resistance memories transmitted by these markers strongly benefit the formation of adaptability against fungicidal stress. The elevation of EC_50_ and drop in 6mA levels quickly reverted to normal levels after fungicidal stress was relieved (Fig. 7F, G, and K). Surprisingly, successively cultivating JA8 on SYP-14288-amended plates for 10 generations resulted in the resistance elevation against all tested fungicides belonging to different MoAs (Fig. 7L), which further confirms that the persistent uncoupler stress could trigger MFR. Overall, these data demonstrated that 6mA is a dynamic intergenerational marker that modulates multi-fungicidal stress responses and could transmit adaptive advantage to progenies, which enables heightened adaptive potential to stresses in resistant isolates with a rapid and reversible way.

## DISCUSSION

In this study, 6mA was identified as the novel and major DNA marker in *P. capsici* genomes, and it is identified as an essential factor in fungicidal stress response and resistance. 6mA appears to be common in eukaryotic genomes, but its abundance (6mA/A) is as low as 0.00019%–2.8% among different eukaryotes (16). In *P. infestans* and *P. sojae*, the abundance is around 0.05%, determined by MeDIP-seq (15). However, 6mA abundance in *P. capsici* reached 1.67% according to ONT-seq, which is different from that in the two other *Phytophthora* species. This could be caused by genetic differentiation among different *Phytophthora* species and may be attributed to the diversity of sequencing methods (64). ONT-seq is a popular third-generation sequencing technology and is a reliable and widely accepted method in 6mA research (65). The long reads enable an accurate sequencing in ONT-seq.

The distribution pattern of 6mA is important to evaluate its biological significance. Here, DNA 6mA methylome was detected by ONT-seq in *P. capsici* and was evenly distributed across its genome; this is consistent with reports from other organisms (66, 67). The distribution characteristics may indicate that 6mA in *P. capsici* is independent of DNA sequence because no modification enrichment existed in the genome. Interestingly, the methylation marker in RNA could be transmitted to DNA. This further confirms that a portion of 6mA in DNA may be randomly transferred from RNA catabolism and deoxy-nucleotide biosynthesis (68). Furthermore, the majority of 6mA peaks were in the promoter and intergenic regions, which implies that 6mA may contribute to transcription regulation of genes and silencing of intergenic regions. However, it is reported that a partial reduction of 6mA levels resulted in some virulence impairment in *P. sojae* (15). The role of 6mA in *P. capsici* development and virulence, except abiotic stress responses, which are studied here, warrants further investigation.

The relationship between 6mA and gene expression is diverse among different organisms. For example, 6mA depletion is primarily located upstream of transcription start sites in *Chlamydomonas* (15), while it is a negative gene expression mark in mouse embryonic stem cells (18). In *Phytophthora*, it is reported that 6mA modification is primarily associated with lowly expressed genes (15). Here, the hypermethylation in *upr-GSTZ1* resulted in gene overexpression; thus, it could not be concluded whether 6mA is a repressive mark or an active marker in certain organisms. The role could be switched due to the genomic locus and interaction with other cell components or epigenetic modifications of 6mA. Interestingly, mutation of the five predicted methylation sites in *upr-GSTZ1* had no effect on its methylation, while the methylation is absent when the 100 bp sequence near the five predicted sites was truncated. This implies that 6mA writers in *P. capsici* is not site-specific but region-specific. Therefore, further studies on the DNA recognition mechanisms of *Phytophthora* DAMTs are needed. 6mA could interplay with histone H3K4me3 modification, which is further involved in chromatin remodeling and resulted in the change of chromatin accessibility. Future investigations are required to explore the roles of 6mA in spatial and temporal gene expression regulation as well as its crosstalk with other epigenetic modulators. Meanwhile, certain TFs involved in the chromatin binding and gene regulation remain to be studied.

Three *DAMT* genes, but no other N6-adenine methyltransferases, were identified in this study, which coincides with a previous study (15). All three DAMTs in *P. sojae* are required for efficient 6mA methylation and gene expression control (15). Herein, three PcDAMTs demonstrated methyltransferase activity; PcDAMT1 was the main effector in inducing fungicide resistance in *P. capsici*, which implies a functional diversity of the DAMTs. With relative conservation and high expression levels of PcDAMT1, it is the hub effector in *Phytophthora* to handle stress. However, determining the exact roles of the three PcDAMTs in targeting genomic compartments and their functional diversity in *P. capsici* requires further investigation.

In the agricultural system where many pesticides are applied, pests require strategies to rapidly and efficiently adapt their metabolisms and, thus, eventually developed unique genetic characteristics and growth features (69). However, there is no clear evidence about the importance of DNA methylation in pesticide resistance in pests or plant pathogens. The present study attempted to explore whether the association exists in agricultural pests. This study revealed that global DNA hypomethylation is preferentially found in SYP-14288-resistant *P. capsici*, indicating that the transcriptional changes of diverse pathways, including antibiotic biosynthesis, oxidative phosphorylation, glycolysis, and tricarboxylic acid (TCA) cycle could contribute to resistance by 6mA modification alterations. Unlike the resistance mediated by target mutation or metabolism, the epigenetic study provides a more complex model in which resistance developed. Overall, these results underscore the significant role of global DNA methylation and a specific regulatory axis in resistance development, enriching our current understanding of pesticide resistance at the molecular level.

Uncouplers are a unique type of MIs, and they lack specific target proteins like other fungicides (3). In the agricultural field, fluazinam is the most popular uncoupler, which can control almost all plant pathogens including fungi, oomycetes, bacteria, and nematodes (45). With intense application, uncoupler resistance emerged in some species, including *Botrytis cinerea*, *P. infestans,* and some bacteria (63, 70, 71), but the molecular basis of the resistance may be triggered by P450s, GSTs, or transporters, respectively (47, 72–75). Herein, DAMT1 and DNA 6mA as the upstream regulator and the crucial factor induced GSTZ1-mediated uncoupler resistance (76, 77).

We have surprisingly found here that SYP-14288-resistant isolates showed cross-resistance with multiple fungicides, especially the MIs; this coincides with a previous study reporting that large changes in mitochondrial features occurred in SYP-14288-resistant isolates (3). The “low energy shock’’ adaptive response in uncoupler-resistant bacteria (63) is consistent with the current finding that mitochondrial function is damaged in RJA1, which further implies that less energy is demanded in uncoupler-resistant organisms. Thus, RJA1 could be resistant to multiple fungicides, especially against MIs that inhibit energy production. Mitochondrial damage in RJA1 could result in an ROS burst, leading to transcriptional profile changes in resistant isolates. The excess production of ROS in resistant isolates could also be alleviated by cellular processes associated with ferroptosis, such as enhanced GST activity and reduced GSH abundance. ROS is also well-known for its function as a signaling molecule to affect transcription by modulating the expression of some transcription factors, including ATFS1. In worms, a mitochondria-to-nucleus communication through ATFS1 was initiated after mitochondrial dysfunction (78), which activated the expression of stress response genes to buffer the mitochondrial protein-folding environment and reset the metabolic state (18). Therefore, the expression change of 6mA modifier DMAT1/DAMT3 may also be under the control of the transcriptional stress response. Herein, the mitochondrial damage occurred in SYP-14288-resistant isolates, which could be a consequence of long-term mitochondrial stress imposed by uncouplers, and the biochemical changes in resistant cells, induced by the damage, should be a compensatory effect to adapt to the stresses. Moreover, Ma et al. demonstrated that DAMT1 is the methyltransferase that responds to mitochondrial stress (26). Consistent with this study, PcDAMT1 is the main effector of uncoupler resistance formation.

It is common for many MFR mutants to sustain fitness penalties (8), but the mechanism of fitness penalty is complicated. Here, ferroptosis in RJA1 caused phenotypic changes in resistant isolates, including weak growth vigor and virulence reduction (3). Cancer cells exhibit a higher dependence on iron than normal cells (79), making them more susceptible to iron-catalyzed necrosis. Clinical drugs like sulfasalazine can induce ferroptosis by modulating iron metabolism and enhancing lipid peroxidation (36, 37, 80). Like these clinical drugs, this study showed that agricultural uncouplers could induce ferroptosis in resistant isolates, and the ferroptosis-related cell death contributed to MFR and fitness penalty. This is possibly due to the low energy consumption state in uncoupler-resistant isolates, which could be more resistant to other environmental stresses. Moreover, ferroptosis-inducer synergism with uncouplers could significantly promote MFR in *P. capsici*, which serves as a reminder of an approach to better control plant diseases that is similar to the clinical chemotherapy against cancer cells by inhibiting ferroptosis (35). The mapping of 6mA modification is changed after persistent fungicide application, and the change is accompanied by a sensitivity reduction against the fungicide in SYP-14288-resistant isolates. Interestingly, a common occurrence in this study was that the insensitivity against fungicides was quickly lost when the sequential exposure of plant pathogens to fungicides was stopped. This implies that 6mA could be an effective intergenerational inheritance of stress adaptation in low genomic 6mA background, and the marker could enable *Phytophthora* to adapt more quickly to multiple fungicidal stresses. However, the marker was not maintained when the fungicidal stress was removed, which supports the finding in *C. elegans* that reversal of mitochondrial stress adaptation in later generations is due to epigenetic markers that may only be inherited for a limited number of generations (26).

**Fig 8:**
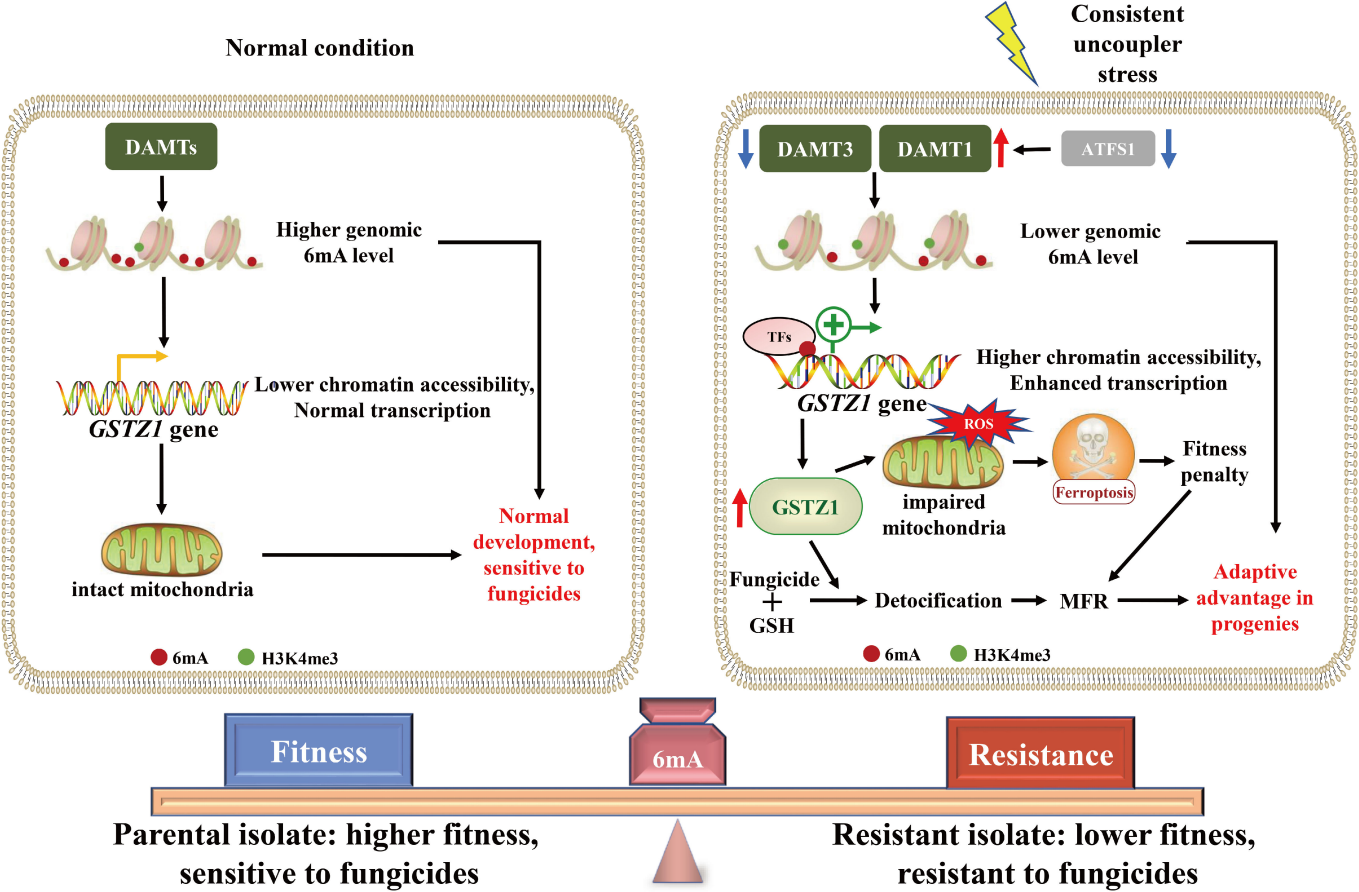
DNA 6mA confers the trade-off between fitness and fungicide resistance. DNA 6mA is an important epigenetic marker which is involved in abiotic stress response in *Phytophthora capsici*. In wild-type isolate, 6mA and H3K4me3 levels are higher and lower compared with resistant isolate, respectively. The stable methylation states in different generations result in a normal development state, which shows high fitness but remains sensitive to fungicides (left panel). Otherwise, consistent exposure to uncoupler (e.g., SYP-14288) could induces huge changes in the epigenetic pathway and enables MFR to *P. capsici*. DAMTs with altered expression levels lead to a relatively low genomic DNA level (also a relatively high H3K4me3 level) in resistant isolates. Furthermore, a *zeta-GST gene*, *GSTZ1,* is overexpressed due to the 6mA modifications located in its promoter region and the elevated chromatin accessibility. The overexpression of GSTZ1 induces mitochondrial damage, triggers the eruption of reactive oxygen species (ROS), and ultimately leads to ferroptosis. This cascade of events results in a fitness penalty observed in resistant isolates. GSTZ1 directly participates in detoxification by catalyzing the complex formation between fungicides and GSH. Its overexpression, combined with ferroptosis, significantly enhances resistance to various fungicides in *P. capsici*. Meanwhile, lower genomic 6mA level enables effective intergenerational inheritance of stress adaptation in resistant isolates, which cuts down the fitness but enables adaptive advantage against multiple fungicides in these isolates. Taken together, 6mA is an important weight to balance fitness and fungicidal resistance; it could also act as an essential transgenerational marker to transmit intergenerational resistance memory and develops a heightened adaptive advantage under stress.

In conclusion, genomic DNA 6mA is modulated by PcDAMT1 and mediate fungicide resistance and resistance inheritance in *P. capsici* (Fig. 8). PcDAMT1-mediated PcGSTZ1, an innovative mechanism, is the key to cause resistance through increasing the metabolism of SYP-14288 and inducing ROS burst, mitochondrial damage, and ferroptosis. ROS may act as a feedback regulator to control the 6mA modification. Furthermore, the 6mA-GSTZ1-ferroptosis axis may promote the formation of a low energy consumption state, which contributes to the trade-off between MFR and fitness. Simultaneous application of uncoupler fungicides with different MoA could confer rapid MFR formation. Thus, solely applied uncouplers should be advocated. To our knowledge, this is the first time reporting the regulation of MFR and fitness penalty by 6mA and ferroptosis. The results provide new ideas for further research on the molecular basis of multiple fungicides/drugs resistance, the function of 6mA in eukaryotes, and the proper application of fungicides.

## Data Availability

All data supporting the findings of this study are available within the paper and within its supplemental material. Raw sequences for RNA-seq and ONT-seq have been made publicly available at NCBI SRA (accession codes PRJNA940213 and PRJNA940278). Source data are provided with this paper.

## SUPPLEMENTAL MATERIAL

File S1 mbio.03177-23-s0001.docx The 176 DEGs with DMLs in JA8 vs RJA1.

File S2 mbio.03177-23-s0002.xlsx bZIP proteins in *P. capsici*.

Supplemental material mbio.03177-23-s0003.xlsx Tables S1 to S3; captions for Files S1 and S2; Figures S1 to S14.

ASM does not own the copyrights to Supplemental Material that may be linked to, or accessed through, an article. The authors have granted ASM a non-exclusive, world-wide license to publish the Supplemental Material files. Please contact the corresponding author directly for reuse.
