# Supplementary material for: N6-methyloxyadenine-mediated detoxification and ferroptosis confer a trade-off between multi-fungicide resistance and fitness
Source: mBio. 2024 Jan 31;15(3):e03177-23. doi: 10.1128/mbio.03177-23 (PMC10936191; doi:10.1128/mbio.03177-23)
Supplement: File S1 — The 176 DEGs with DMLs in JA8 vs RJA1. [file mbio.03177-23-s0001.docx]

**Supplementary Information**

Table S1. Differentially expressed genes related to glutathione metabolism in JA8 vs RJA1.

Table S2. Primers list.

Table S3. Sensitivity of JA8 and RJA1 against commercial fungicides belong to different mode of actions.

Supplementary file1. The 176 DEGs with DMLs in JA8 vs RJA1.

Supplementary file2. Mitochondrial targeting signal (MTS) analysis of all bzip transcription factors in *Phytophthora capsici*.

Figure S1. Chemical structures of SYP-14288 (A) and its analogue compound Fluazinam (B).

Figure S2. DNA 5-methylcytosine (5mC) modification is absent in *Phytophthora capsici*.

Figure S3. *In vitro* expression of 3×FALG-tagged PcDAMTs.

Figure S4. Transcriptome analysis of RJA1 and JA8 with or without SYP-14288 treatment.

Figure S5. Cluster analysis of the differentially expressed genes (DEGs) in JA8 vs RJA1 and their gene ontology analysis.

Figure S6. Classification of all expressed genes in JA8 vs RJA1 according to their expression profiles.

Figure S7. Screening of genes associated with SYP-14288 resistance discovered by RNA-seq and ONT-seq.

Figure S8. Confirmation of 3×FLAG tagged PcDAMTs expression in *Escheria coli*.

Figure S9. Expression level of *PcDAMT1* in RJA1 and RJA1/siDAMT1 (A) and *PcDAMT3* in LT1534 and LT/siDAMT3 (B).

Figure S10. Confirmation of the expression of 3×FLAG tagged *PcGSTZ1* in *Escheria coli* (A) and *Phytophthora capsici* (B).

Figure S11. Confirmation of the transformation of SYP-14288 to SYP-14288-GSH by absorption photometry.

Figure S12. Relative gene expression level of *PcATFS1* in SYP-14288-resistant mutants or their parental isolate (A) and before or after H_2_O_2_ treatment (B), the relationship of gene expression level between *PcATFS1* and *PcDAMT1*/*PcDAMT2*/*PcDAMT3*/*PcGSTZ1* (C).

Figure S13. Characterization of ferroptosis in SYP-14288-resistant isolates and PcGSTZ1 overexpression mutant.

Figure S14. A glutathione peroxidase gene *PcGPx1* was involved in SYP-14288 resistance.

**Table S1. Differentially expressed genes related to glutathione metabolism in JA8 vs RJA1.**

| Gene ID | Description | fpkm in JA8 | fpkm in RJA1 | Fold change (RJA1/JA8) |
| --- | --- | --- | --- | --- |
| 96716 | Glutathione S-transferase | 1.00 | 3.00 | 3.00 |
| 98049 | Glutathione S-transferase | 4.23 | 23.29 | 5.51 |
| 510671 | glutathione transferase | 952.70 | 549.83 | 0.58 |
| 502718 | Glutathione S-transferase | 26.03 | 66.70 | 2.56 |
| 9707 | Glutathione S-transferase | 139.85 | 366.02 | 2.62 |
| 511938 | Glutathione S-transferase | 318.00 | 1125.96 | 3.54 |
| 109612 | Glutathione S-transferase | 16.86 | 9.56 | 0.57 |
| 545413 | Glutathione S-transferase | 3.61 | 8.03 | 2.23 |
| 121496 | Predicted glutathione S-transferase | 22.21 | 32.21 | 1.45 |
| 125626 | Predicted glutathione S-transferase | 8.70 | 22.48 | 2.58 |
| 545222 | Predicted glutathione S-transferase | 326.61 | 63.71 | 0.20 |
| 553737 | Glutathione S-transferase | 25.51 | 55.75 | 2.19 |
| 21463 | Glutathione S-transferase | 32.22 | 45.62 | 1.42 |
| 511743 | Glutathione S-transferase | 18.41 | 26.26 | 1.43 |
| 41396 | Glutathione peroxidase | 3.49 | 8.25 | 2.36 |
| 503294 | Glutathione peroxidase | 120.27 | 383.76 | 3.19 |
| 510931 | Glutathione peroxidase | 51.09 | 72.92 | 1.43 |
| 557952 | Glutathione peroxidase | 21.67 | 78.85 | 3.64 |
| 503727 | Glutathione peroxidase | 647.79 | 2060.89 | 3.18 |

Note: the two genes marked with red color are the only two genes both differentially expressed and methylated in JA8 vs RJA1, other genes are only differentially expressed.

**Table S2. Primer list**

| Primer ID | Sequence (5’-3’) | Destination |
| --- | --- | --- |
| DAMT1F | ATGGAGGCCCTACTTTCCAG | amplification of *PcDAMT1* |
| DAMT1R | CTACTCCTCCTCATCCGA |  |
| DAMT2F | ATGGACGAATTGCTAGCGA | amplification of *PcDAMT2* |
| DAMT2R | CTATTCATCCTTCTCGTCGG |  |
| DAMT3F | ATGAAGGACCGAAGCGCAA | amplification of *PcDAMT3* |
| DAMT3R | CTATTCGAAGTCTACTTCTGGGTT |  |
| DamF | ATGAAGAAAAATCGCGCTTTTT | amplification of *EscDam* |
| DamR | TTATTTTTTCGCGGGTGAAACGA |  |
| GFPF | ATGGTGAGCAAGGGCGAG | amplification of *GFP* |
| GFPR | TTACTTGTACAGCTCG |  |
| qDAMT1F | ATGCTGACTTGGGCTTCAC | quantification of *PcDAMT1* |
| qDAMT1R | AGTCCTCTGTGCTCTCACCA |  |
| qDAMT2F | AAGGAGATGAAGGCGGACT | quantification of *PcDAMT2* |
| qDAMT2R | AACCGCTCCGACTCGTAAT |  |
| qDAMT3F | AAGAGAACGCAGCGATGA | quantification of *PcDAMT3* |
| qDAMT3R | CTTCTGGGTTTACAGCATTGAG |  |
| siDAMT1F | CTACTCCTCCTCATCCGA | gene silencing of *PcDAMT1* |
| siDAMT1R | ATGGAGGCCCTACTTTCCAG |  |
| siDAMT3F | CTATTCGAAGTCTACTTCTGGGTT | gene silencing of *PcDAMT3* |
| siDAMT3R | ATGAAGGACCGAAGCGCAA |  |
| GSTZ1F | ATGGCGACTAAAGAGCAGTACG | amplification of *PcGSTZ1* |
| GSTZ1R | TTAATTGACTGCGTCAGGTTGTTG |  |
| qGSTZ1F | AGAAGGAATTGGGCAAGACT | quantification of *PcGSTZ1* |
| qGSTZ1R | ATTGACTGCGTCAGGTTGTTG |  |
| uprF | TGCACGTCGTCCAATTTTCAT | amplification of *upr-GSTZ1* |
| uprR | GGGCTTATAACGAAGGAAGG |  |
| CKuprF | ATGGCGACTAAAGAGCAGTA | amplification of the control sequence near *upr-GSTZ1* |
| CKuprR | TCCTCACTTCGAGCCGTG |  |
| qDIPuprF | GCGTCTCCTCAGCGTAATTC | 6mA-DIP-qPCR of *upr-GSTZ1* |
| qDIPuprR | TACAGACGTGGACGACAAGC |  |
| qDIPCKuprF | CAGTCCAGTGCAATCCTTGA | 6mA-DIP-qPCR of control sequence |
| qDIPCKuprR | TCTGTTGCCTTGACTTGCAC |  |
| muuprF1 | TGCACGTCGTCCAATTTTCAT | amplification of *mu-upr-GSTZ1*, fusion PCR |
| muuprR1 | AACCAAAGGACACCCCAGGTG |  |
| muuprF2 | CACCTGGGGTGTCCTTTGGTT |  |
| muuprR2 | GGGCTTATAACGAAGGAAGG |  |
| truuprF1 | TGCACGTCGTCCAATTTTCAT | amplification of *tru-upr-GSTZ1*, fusion PCR |
| truuprR1 | GAGGAGGACGGAGATTTGGC |  |
| truuprF2 | GCCAAATCTCCGTCCTCCTC GATTGCGCATCGTCTTCCTC |  |
| truuprR2 | GGGCTTATAACGAAGGAAGG |  |
| qGFPF | CATCTGCACTACCGGAAAGC | quantification of *GFP* |
| qGFPR | ACGTGTCTTGTAGTTCCCGT |  |
| qATFS1F | GCAAGATCGTTGAGGAGA | quantification of *PcATFS1* (508997) |
| qATFS1R | TCCGTTTGTCCGATAATC |  |
| GPx1F | ATGCTGGTCGGTCGGGTG | amplification of *PcGPx1* (41396) |
| GPx1R | CTACTTGTATCGTCCGTCGTC |  |
| 556060F | ATGGCGGAAGTGTCAGCCTATT | amplification of 556060 |
| 556060R | TTAGTTGGACACGCTGCTGG |  |
| 539682F | ATGGTGGGCGTGGCAATGAA | amplification of 539682 |
| 539682R | TCATACCGGTGTGCGATAGCTC |  |
| 21549F | ATGTGGACGATCAGTCAGTA | amplification of 21549 |
| 21549R | TTAGCTGCTTGCAGCAGC |  |
| 111818F | ATGTGCACGGCCAGCGGCT | amplification of 111818 |
| 111818R | CTACACTTCCTCTTTTCGCGGTAAA |  |
| qGPx1F | AGAAGTACAGTGAAAAGGGTCTTA | quantification of *PcGPx1* |
| qGPx1R | GTGCTGTATTTGTCCGCAATC |  |
| qMITOF | GCAAGATGTTCACCTATGTTTGA | quantification of mitochondrion (*NADH: Ubiquinone oxidoreductase core subunit 5* gene is used as the reference) |
| qMITOR | TCTTGTTCGTCTCCCATTGC |  |
| qNUCLF | GGGAGGTTTGGGAGCTCTAC | quantification of nucleus (*PcDAMT3* is used as the reference) |
| qNUCLR | TCGCTGCGTTCTCTTCTACA |  |
| qWS21F | GGAAAGAACAAACGCCTGAC | quantification of the internal reference gene *PcWS21* |
| qWS21R | GTTGCGCTCCGAGAAGATA |  |

**Table S3. Sensitivity of JA8 and RJA1 against commercial fungicides belong to different mode of actions.**

| Category^a^ | Fungicide | Isolate | Equation^b^ | Correlation^c^ | EC_50_ (μg/mL) ^d^ | RF^e^ |
| --- | --- | --- | --- | --- | --- | --- |
| MI^f^ (uncoupler) | Fluazinam | JA8^g^ | Y=4.8954+0.74X | 0.9071 | 1.39 |  |
|  |  | RJA1 ^h^ | Y=4.0961+0.60X | 0.9039 | 31.44 | 22.62 |
| MI (Quinone outside inhibitor, QoI) | Azoxystrobin | JA8 | Y=4.4432+0.78X | 0.9755 | 5.21 |  |
|  |  | RJA1 | Y=3.6119+1.06X | 0.9747 | 20.28 | 3.89 |
| MI (Quinone inside inhibitor, QiI) | Cymoxanil | JA8 | Y=4.1088+0.39X | 0.9764 | 76.50 |  |
|  |  | RJA1 | Y=3.8531+0.98X | 0.9825 | 23.21 | 0.30 |
| Cellulose synthetase inhibitor | Dimethomorph | JA8 | Y=6.6948+3.62X | 0.9978 | 0.34 |  |
|  |  | RJA1 | Y=6.0931+2.78X | 0.9896 | 0.40 | 1.18 |
| Oxidized sterol binding protein inhibitor | Oxathiapiprolin | JA8 | Y=18.9996+4.36X | 0.9936 | 0.000614 |  |
|  |  | RJA1 | Y=13.3075+3.04X | 0.9415 | 0.001869 | 3.04 |
| β-tubulin inhibitor | Zoxamide | JA8 | Y=5.9692+1.03X | 0.9850 | 0.11 |  |
|  |  | RJA1 | Y=5.5989+1.27X | 0.9798 | 0.34 | 3.09 |
| Spectrin inhibitor | Fluopicolide | JA8 | Y=6.1410+1.86X | 0.9998 | 0.24 |  |
|  |  | RJA1 | Y=4.6431+1.15X | 0.8816 | 2.04 | 8.50 |
| RNA polymerase I inhibitor | Metalaxyl | JA8 | Y=4.3968+1.70X | 0.9775 | 2.26 |  |
|  |  | RJA1 | Y=4.7593+0.84X | 0.9890 | 1.94 | 0.86 |
| Multiple-site inhibitor | Chlorothalonil | JA8 | Y=4.5157+0.88X | 0.9919 | 3.55 |  |
|  |  | RJA1 | Y=4.5318+0.38X | 0.5691 | 17.64 | 4.97 |
| Unknown mode of action | Cyazofamid | JA8 | Y=3.9375+1.10X | 0.9964 | 9.29 |  |
|  |  | RJA1 | Y=4.7681+0.53X | 0.9662 | 2.75 | 0.30 |

^a^The mode of action was divided according to the modes of action of fungicide based on FRAC.

^b^Equation: the inhibition rate was transformed to probability value (Y), concentrations of the fungicide were transformed to logarithm (X), then linear regression equation (Y=a+bX) was fit. The logarithm value of X was calculated according to the regression when Y=5. This logarithm value of X was the EC_50_ value.

^c^Correlation between the probability values transformed by inhibition rates relative to the control (solvent only) and different concentrations (log-transformed) of the fungicide(1).

^d^EC_50_, half maximal effective concentration.

^e^RF, resistance factor; ratio of EC_50_ of RJA1 relative to the EC_50_ of JA8.

^f^MI, Mitochondrial inhibitor.

^g^JA8, wild-type *Phytophthora capsici* isolates.

^h^RJA1, SYP-14288-resistant *Phytophthora capsici* isolates.


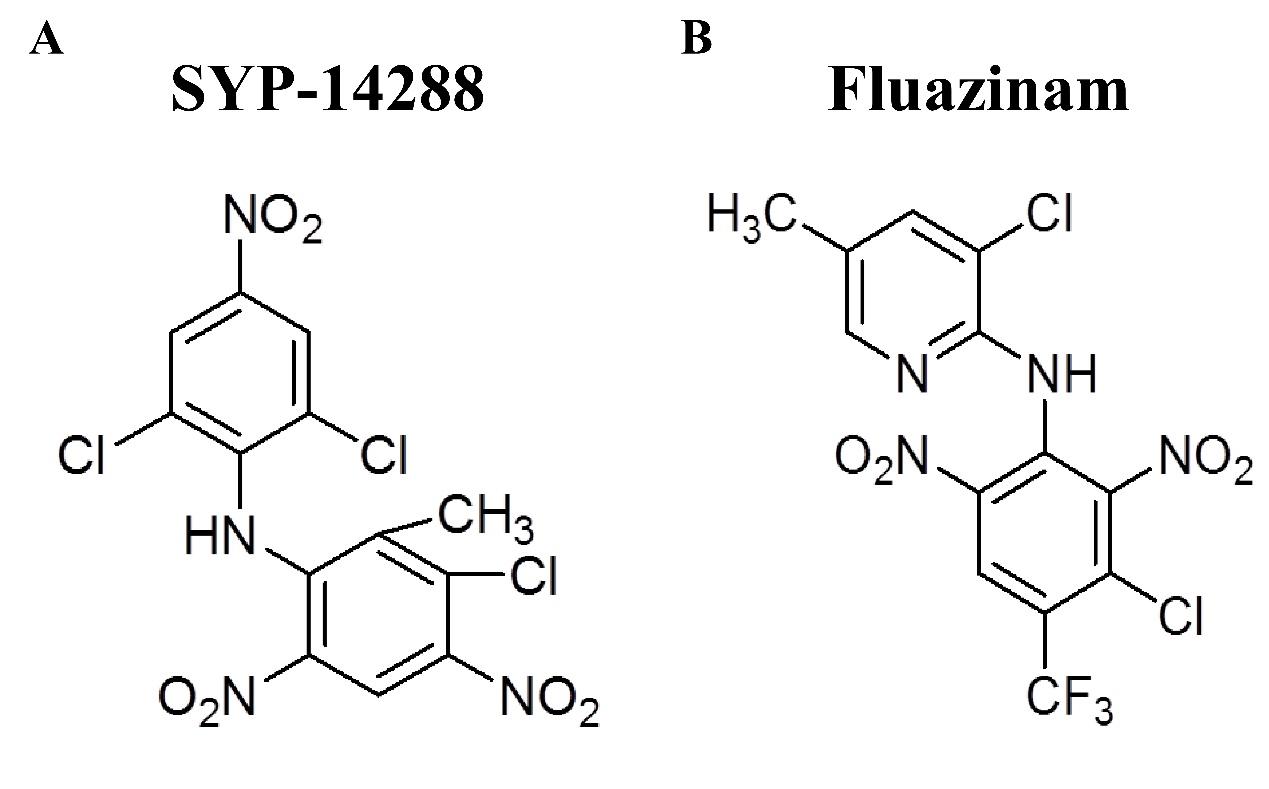


**Figure S1. Chemical structures of SYP-14288 (A) and its analogue compound Fluazinam (B).**


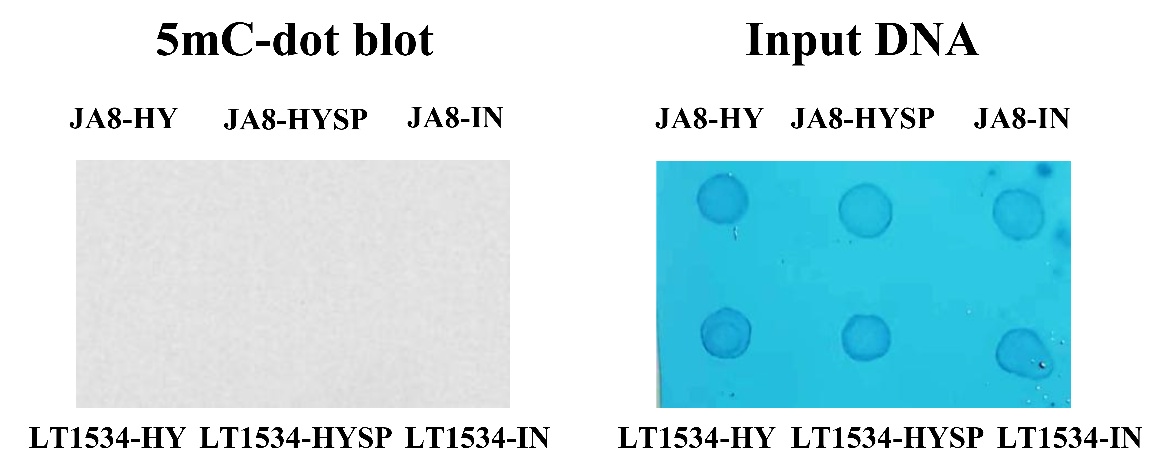


**Figure S2. DNA 5-methylcytosine (5mC) modification is absent in *Phytophthora capsici*.** Genomic DNA was extracted from *P. capsici* in different life stages (HY: hypha; HYSP: sporulated hypha; IN: infection stage) and subjected to dot-blot using a commercial DNA 5mC antibody. Input DNA was treated with 0.1% methylene blue solution and every dot loaded 100 ng DNA. The experiments were independently carried out in three replicates.


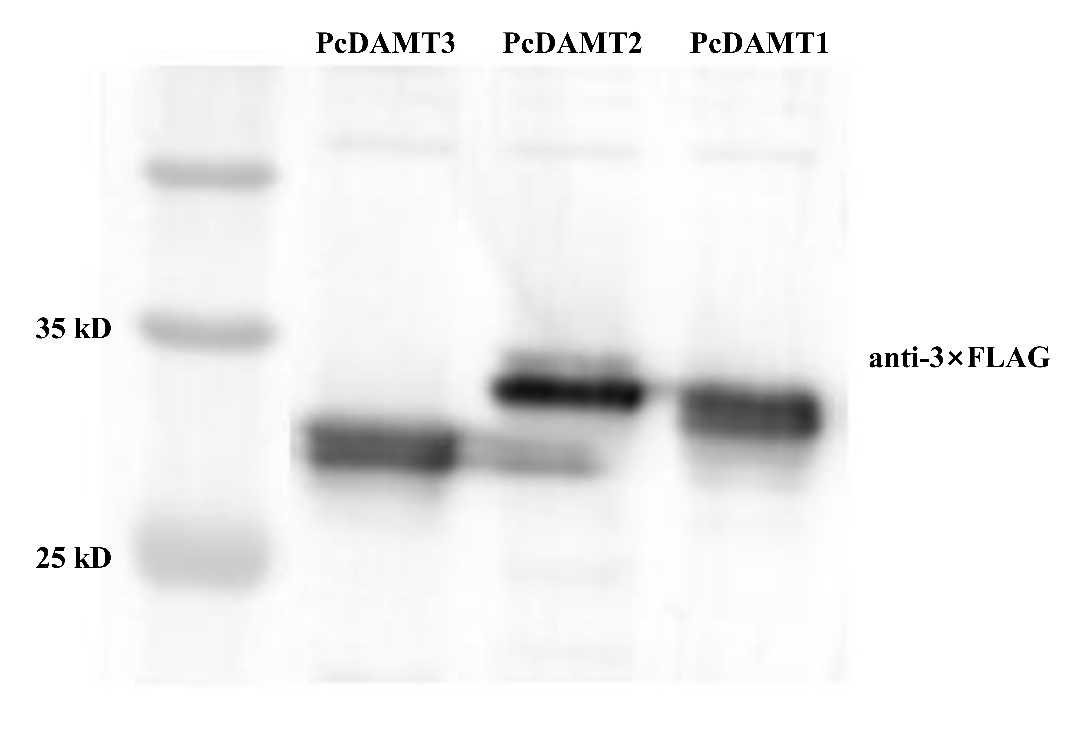


**Figure S3. *In vitro* expression of 3×FALG-tagged PcDAMTs.** Proteins were produced in TNT^®^ SP6 High-Yield Wheat Germ Protein Expression System.


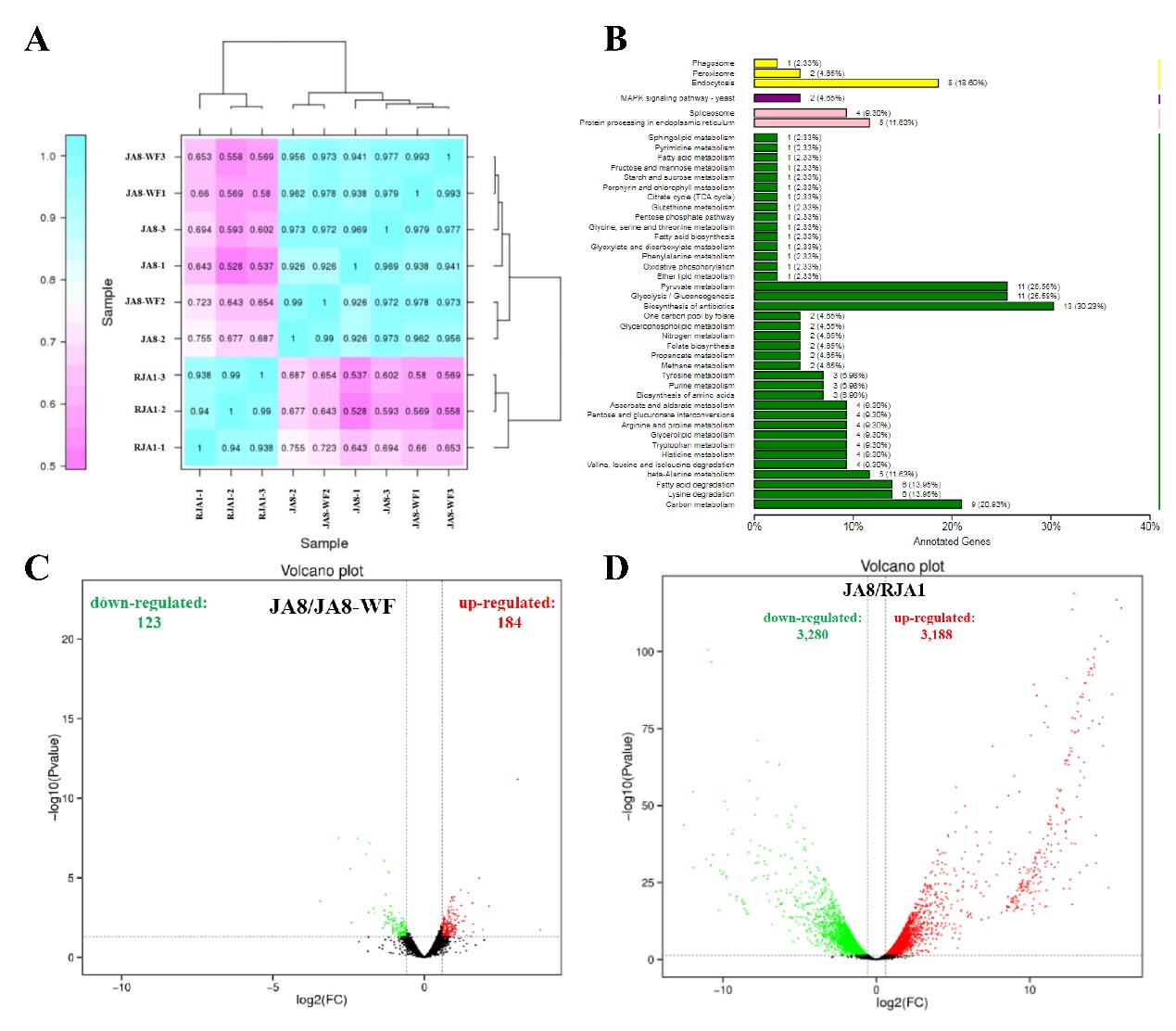


**Figure S4. Transcriptome analysis of RJA1 and JA8 with or without SYP-14288 treatment.** (A) Correlation assessment of samples. The Pearson correlation coefficient R was calculated and acted as an evaluation index of correlation between samples. (B) KEGG annotation of differentially expressed genes in JA8 vs JA8-WF. (C and D) Volcano plot for displaying differential expressed genes between JA8 and JA8-WF (C) or JA8 and RJA1 (D). EdgeR was used for identifying differentially expressed genes.

**
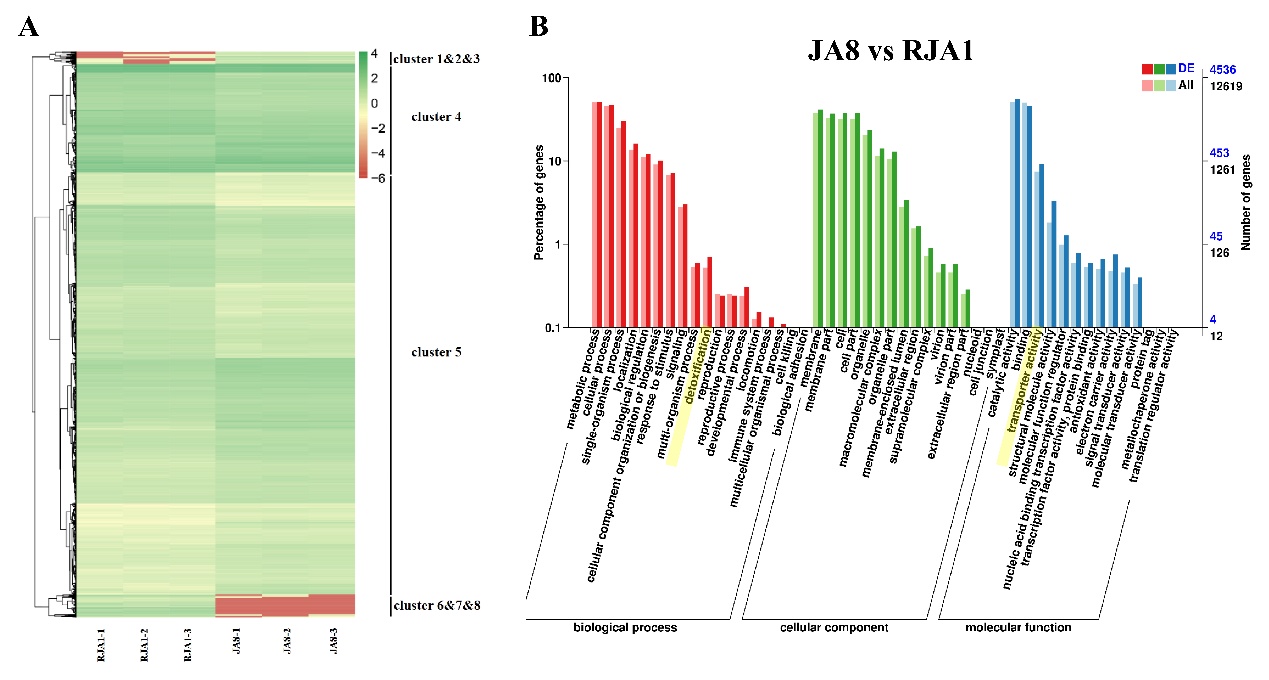
**

**Figure S5. Cluster analysis of the differentially expressed genes (DEGs) in JA8 vs RJA1 and their gene ontology analysis.** (A) Clustering analysis of differentially expressed genes in JA8 vs RJA1. (B) The total genes and the DEGs belong to each classification were labelled with light color and normal color, respectively. Detoxification process and function respected to transporter activity which were highlighted were the two major pathways involved in fungicidal resistance.


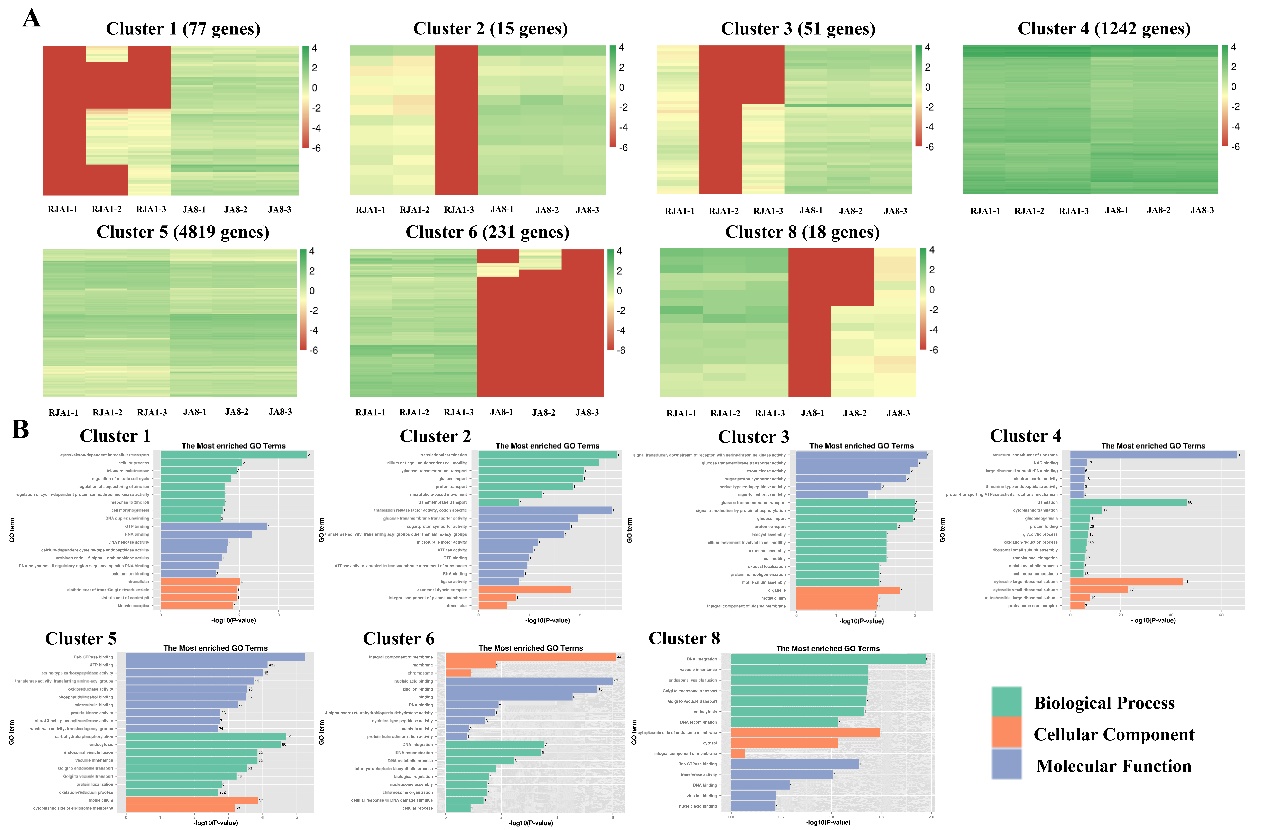
**Figure S6. Classification of all expressed genes in JA8 vs RJA1 according to their expression profiles.** (A) Totally eight clusters were classified, the gene numbers in each cluster were marked in brackets. Green and red represented the highly expressed genes and lowly expressed genes in each sample, respectively. (B) Gene ontology analysis of the genes from each cluster. The cluster 7 are shown in Figure 2 thus only cluster 1 to 5 and cluster 6/7 are shown here.


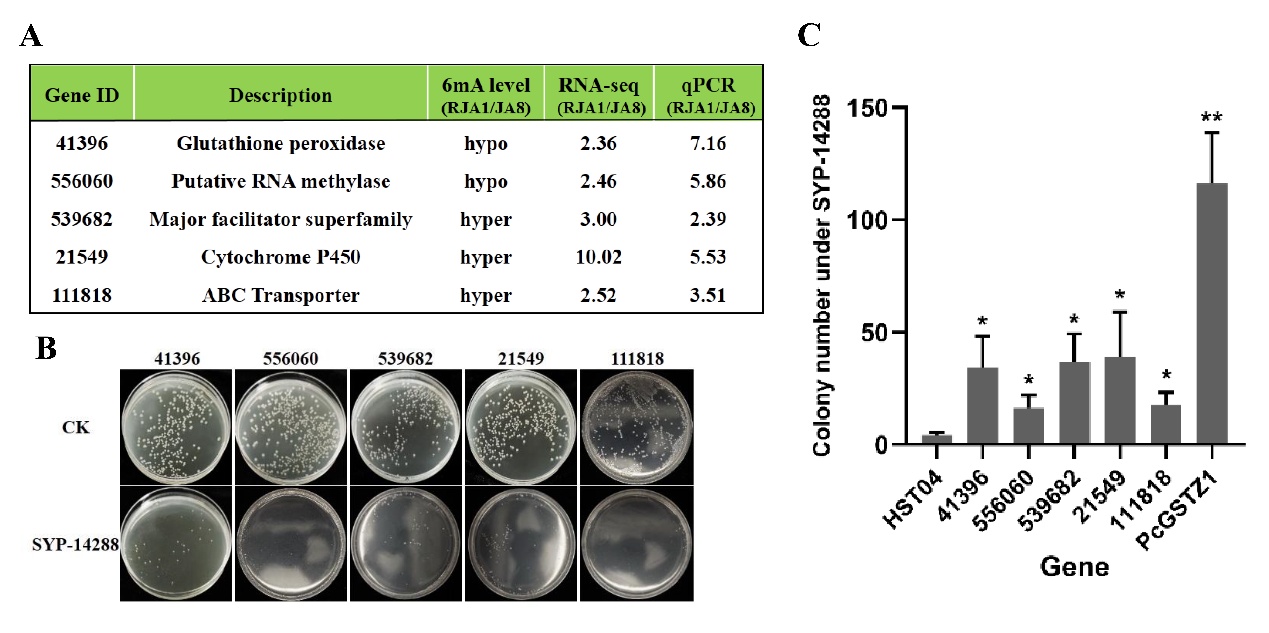


**Figure S7. Screening of genes associated with SYP-14288 resistance discovered by RNA-seq and ONT-seq.** (A) Information of the genes annotated as genes related to drug metabolism or transportation and were both differentially expressed and methylated in JA8 vs RJA1 among the 176 genes. (B) The five genes were expressed in *Escheria coli* respectively and the sensitivity of the mutants against SYP-14288. Colonies were photographed at 24 hpi (hour post inoculation). (C) Colony number of each strains grown on LB plate containing 10 μg/mL SYP-14288. The colony number of *PcGSTZ1*-overexpression *E. coli* strain was used as a positive control, which reflected that PcGSTZ1 is the most efficient gene for SYP-14288 resistance through screening by high-throughput sequencing. The data was Mean ± SD and representative of 3 independent experiments. Statistic analysis by unpaired Student’s t test. * *p* < 0.05; ** *p* < 0.01.


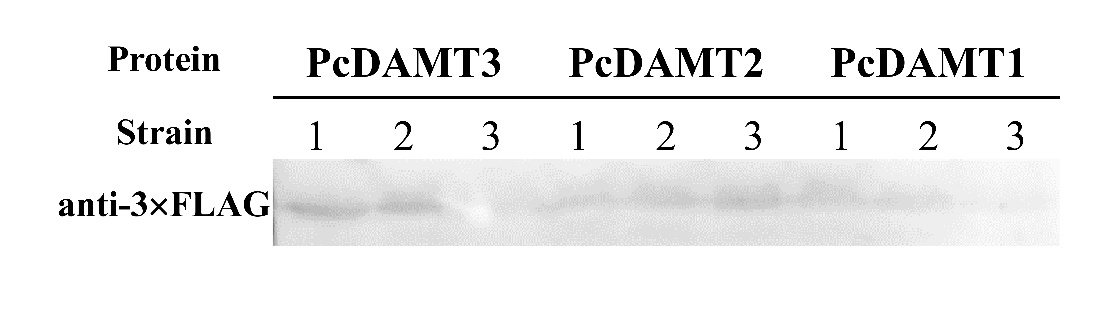


**Figure S8. Confirmation of 3×FLAG tagged PcDAMTs expression in *Escheria coli*.**


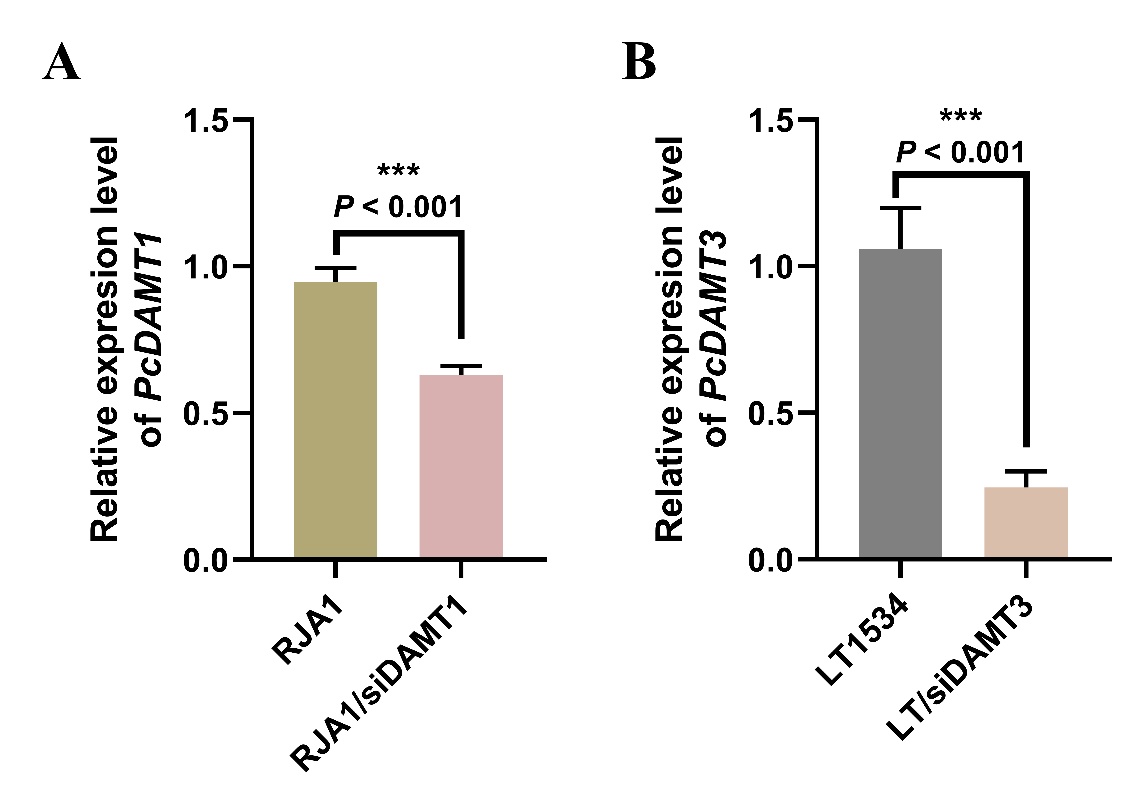


**Figure S9. Expression level of *PcDAMT1* in RJA1 and RJA1/siDAMT1 (A) and *PcDAMT3* in LT1534 and LT/siDAMT3 (B).** *PcWS21* gene was used as an internal reference gene. The data was Mean ± SD and representative of 3 independent experiments. Statistic analysis by unpaired Student’s t test. *** *p* < 0.001.


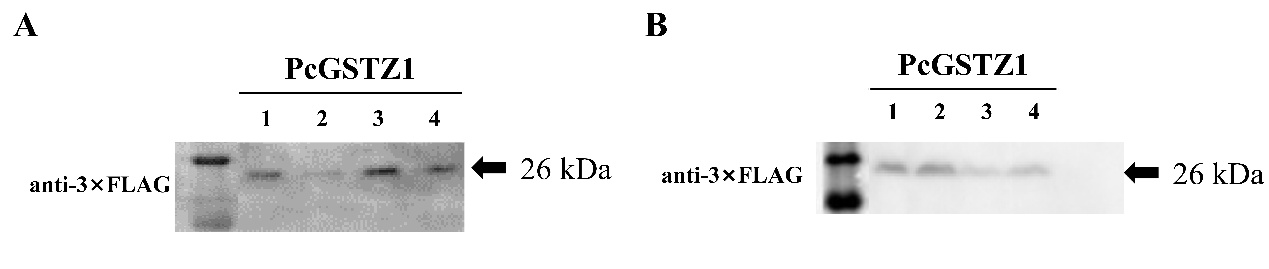


**Figure S10. Confirmation of the expression of 3×FLAG tagged *PcGSTZ1* in *Escheria coli* (A) and *Phytophthora capsici* (B).**


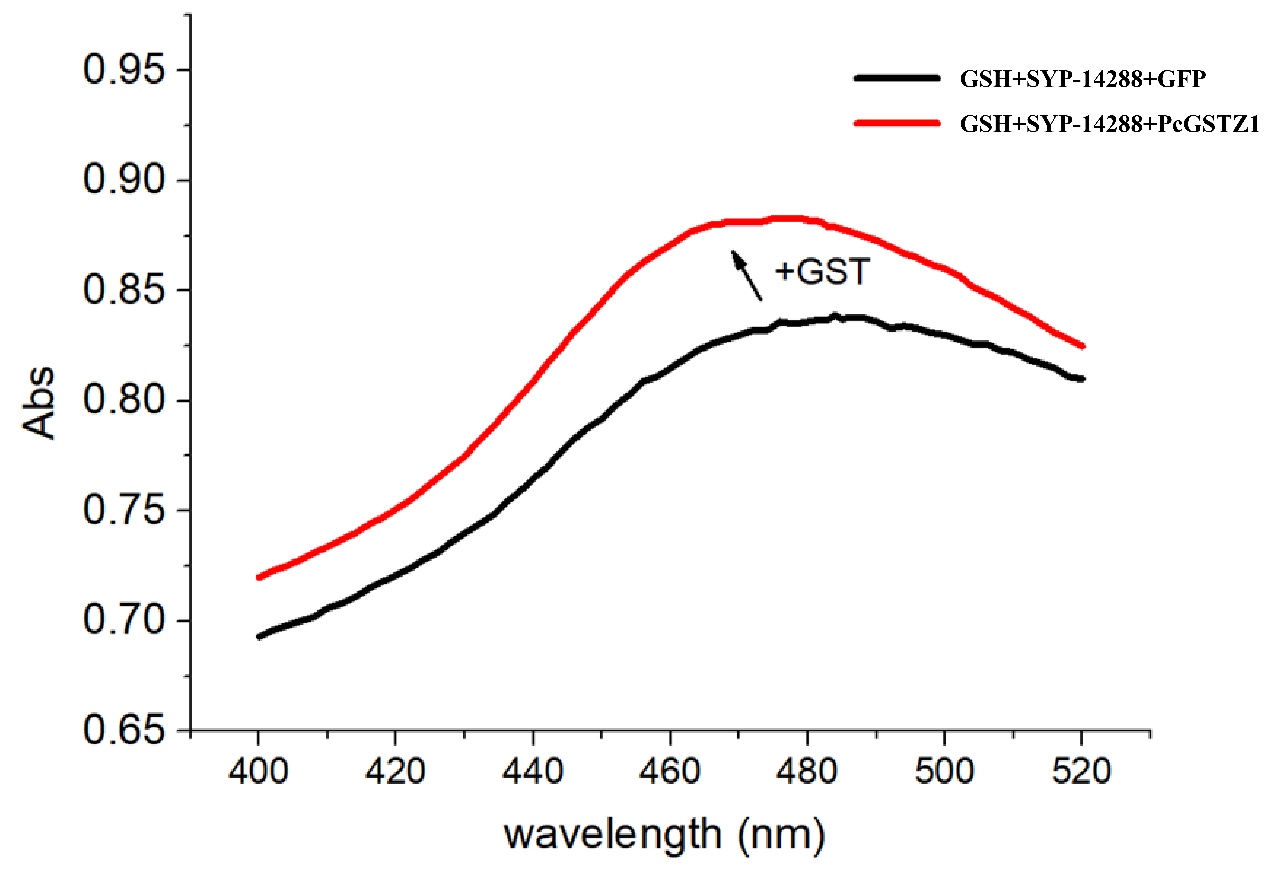


**Figure S11. Confirmation of the transformation of SYP-14288 to SYP-14288-GSH by absorption photometry.** After incubation with GSH and PcGSTZ1, the maximum absorption wavelength of SYP-14288 was significantly excursed, which indicated that SYP-14288 could coupling with GSH under the treatment of PcGSTZ1. SYP-14288, GSH and GFP were incubated together and the product was set as control.


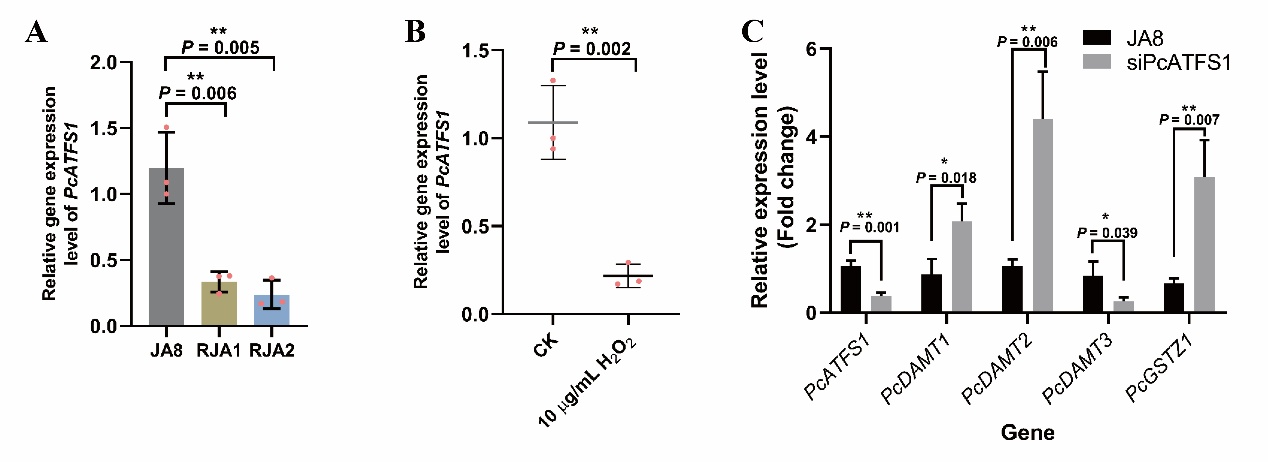


**Figure S12. Relative gene expression level of *PcATFS1* in SYP-14288-resistant mutants or their parental isolate (A) and before or after H_2_O_2_ treatment (B), the relationship of gene expression between *PcATFS1* and *PcDAMT1*/*PcDAMT2*/*PcDAMT3*/*PcGSTZ1* (C).** The data was Mean ± SD and representative of 3 independent experiments. Statistic analysis by unpaired Student’s t test. * *p* < 0.05, ** *p* < 0.01.


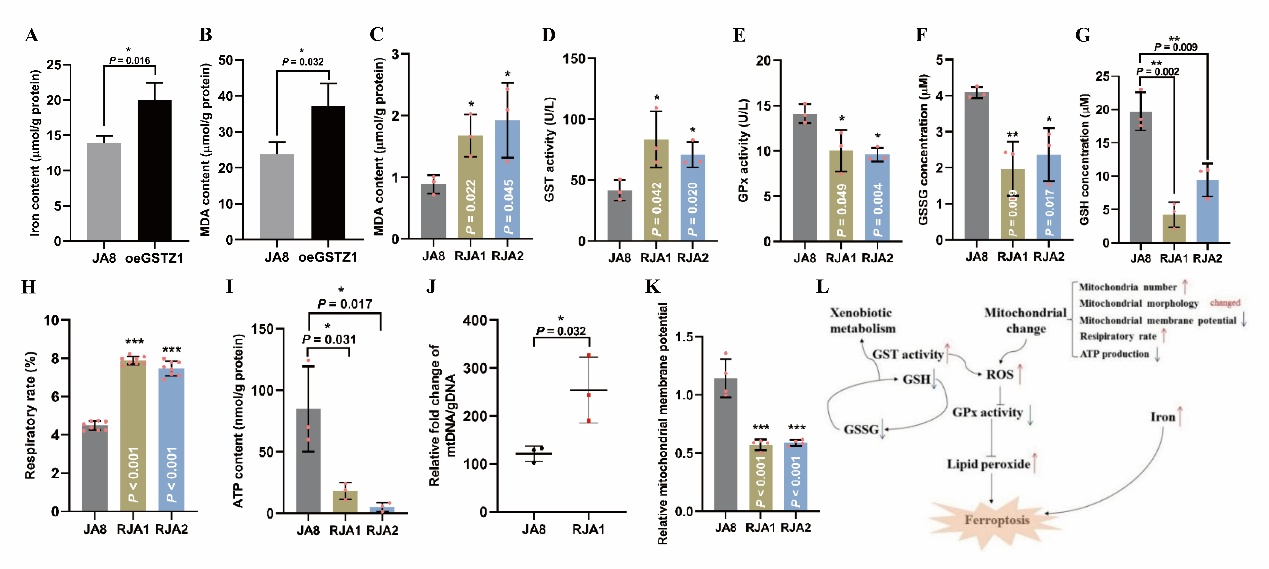


**Figure S13. Characterization of ferroptosis in SYP-14288-resistant isolates and PcGSTZ1 overexpression mutant.** Different landmark characteristics of ferroptosis were significantly changed in oeGSTZ1 and SYP-14288-resistant isolates compared to their parental isolate JA8. Except for iron content shown in Figure 6 and A, MDA content (B and C), GST activity (D), GPx activity (E), GSSG concentration (F), GSH concentration (G), respiratory rate (H), ATP content (I) and mitochondrial membrane potential (K) in the hypha of different isolates was detected by commercial kits according to their respective protocols. For A-C and I, the contents were adjusted by per gram of protein extracted from tissues. (J) Mitochondria number was elevated in RJA1 compared to JA8. Mitochondrial gene *ND1*and nucleus gene *DAMT3* were quantified by qPCR and their relative abundances were used to represent the ratio of mitochondrial DNA (mtDNA) and genomic DNA (gDNA). (L) Schematic illumination of ferroptosis relative characteristics were changed in RJA1 compared with JA8. All data was representative of at least three independent experiments. The data shown in A to K is the mean ± SD. Data was statistically analyzed by unpaired Student’s t test. **P* < 0.05; ***P* < 0.01; ****P* < 0.001.

**
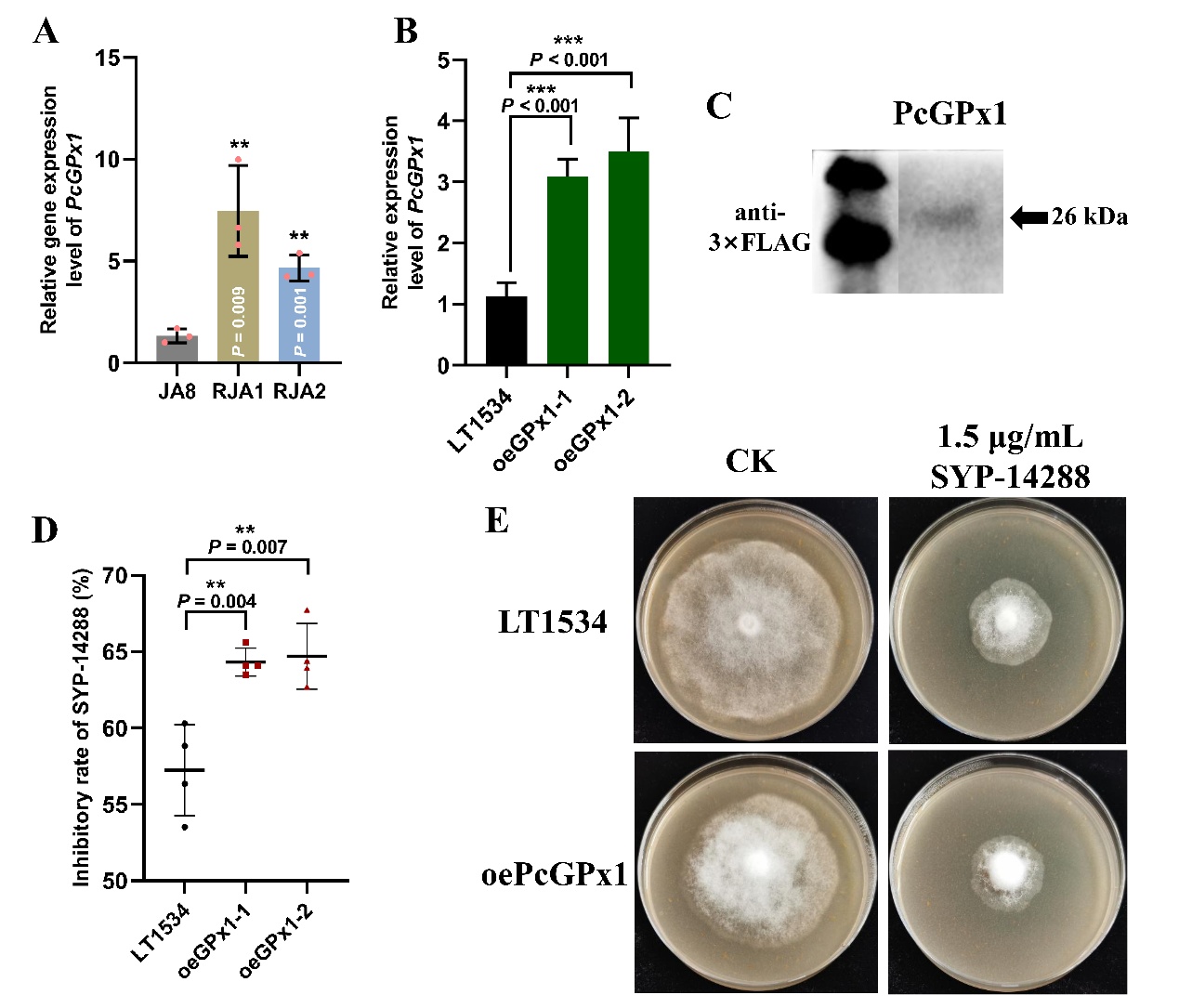
**

**Figure S14. A glutathione peroxidase gene *PcGPx1* was involved in SYP-14288 resistance.** (A and B) Relative gene expression level of *PcGPx1* in SYP-14288-resistant mutants and the *PcGPx1*-overexpression mutants or their respective parental isolates. *PcWS21* gene was used as an internal reference gene. (C) Confirmation of the expression of 3×FLAG tagged PcGPx1 in *Phytophthora capsici*. (D and E) The sensitivity against SYP-14288 was elevated in *Phytophthora capsici* when PcGPx1 is overexpressed. The inhibitory effect of SYP-14288 on wild-type (LT1534) and two PcGPx1 overexpression isolates (oeGPx1-1 and oeGPx1-2) was detected 3 dpi. The colonies were also photographed at 3 dpi. The data shown in A, B and D was Mean ± SD and representative of 3 independent experiments. Statistic analysis by unpaired Student’s t test. * *p* < 0.05; ** *p* < 0.01; *** *p* < 0.001.

**REFERENCES**

1. Wang C, Zhang J, Chen H, Fan Y, Shi Z. 2010. Antifungal activity of eugenol against botrytis cinerea. Trop Plant Pathol 35.
